# A Sub-Micromolar MraY_AA_ Inhibitor with an Aminoribosyl Uridine Structure and a (*S*,*S*)-Tartaric Diamide: Synthesis, Biological Evaluation and Molecular Modeling

**DOI:** 10.3390/molecules27061769

**Published:** 2022-03-08

**Authors:** Martin Oliver, Laurent Le Corre, Mélanie Poinsot, Michaël Bosco, Hongwei Wan, Ana Amoroso, Bernard Joris, Ahmed Bouhss, Sandrine Calvet-Vitale, Christine Gravier-Pelletier

**Affiliations:** 1 Université de Paris, Faculté des Sciences, UMR CNRS 8601, LCBPT, F-75006 Paris, France; martin.cg.oliver@gmail.com (M.O.); laurent.le-corre@u-paris.fr (L.L.C.); melanie.poinsot@u-paris.fr (M.P.); michael.bosco@u-paris.fr (M.B.); hongwei.wan@etu.parisdescartes.fr (H.W.); 2 Unité de Physiologie et Génétique Bactériennes, Centre d’Ingénierie des Protéines, Département des Sciences de la Vie, Université de Liège, Sart Tilman, B4000 Liège, Belgium; amamoroso@ulg.ac.be (A.A.); bjoris@uliege.be (B.J.); 3 Université Paris-Saclay, INSERM U1204, Univ Evry, Structure-Activité des Biomolécules Normales et Pathologiques (SABNP), F-91025 Evry-Courcouronnes, France; ahmed.bouhss@univ-evry.fr

**Keywords:** MraY transferase, inhibitors synthesis, carbacaprazamycin analogs, molecular docking studies, inhibition tests

## Abstract

New inhibitors of the bacterial tranferase MraY are described. Their structure is based on an aminoribosyl uridine scaffold, which is known to be important for the biological activity of natural MraY inhibitors. A decyl alkyl chain was introduced onto this scaffold through various linkers. The synthesized compounds were tested against the MraY_AA_ transferase activity, and the most active compound with an original (*S*,*S*)-tartaric diamide linker inhibits MraY activity with an IC_50_ equal to 0.37 µM. Their antibacterial activity was also evaluated on a panel of Gram-positive and Gram-negative strains; however, the compounds showed no antibacterial activity. Docking and molecular dynamics studies revealed that this new linker established two stabilizing key interactions with N190 and H325, as observed for the highly potent inhibitors carbacaprazamycin, muraymycin D2 and tunicamycin.

## 1. Introduction

**Figure 1:**
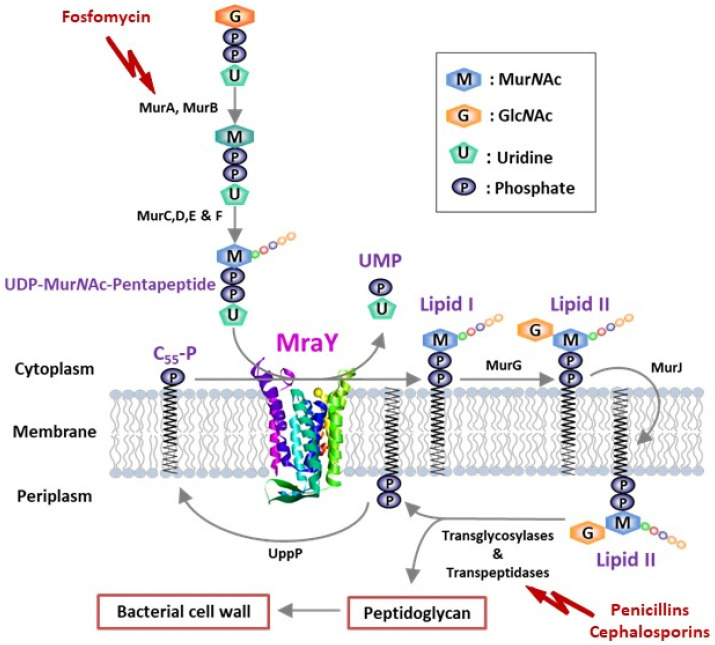
Reaction catalyzed by the bacterial transferase MraY in peptidoglycan biosynthesis.

The continuous emergence of bacterial resistance to commonly used antibiotics is a major risk to global health [1,2,3]. It also has a significant societal impact, particularly in economic terms, due to the increased length of hospital stays because of nosocomial infections that considerably affects hospital costs [4,5,6,7]. Multidrug-resistant (MDR) bacterial strains are capable of developing a set of resistance mechanisms to circumvent the toxicity of antibacterial compounds, and the fight against antimicrobial resistance is a challenge of prime importance for the scientific community. Biological targets displaying a different mode of action than the one targeted by the approved antibiotics are particularly sought after in order to delay the emergence of resistance. Although peptidoglycan biosynthesis is a well-known target, it still involves enzymes specific to the bacterial world that are not targeted by existing drugs and therefore represent promising targets in the search of new antibiotics. Thus, the membrane [8] and intracytoplasmic [9] steps of peptidoglycan biosynthesis are underexploited and deserve special attention. Peptidoglycan biosynthesis is a complex process that takes place successively in the cytoplasm, the membrane and the periplasm (Figure 1). The MraY transferase catalyzes the first membrane-associated step of this biosynthesis, namely the transfer of the phospho-Mur*N*Ac-pentapeptide moiety from the cytoplasmic precursor (UDP-MurNAc-pentapeptide) to the membrane acceptor, undecaprenyl-phosphate (C_55_-P), yielding lipid I and releasing uridine monophosphate [10]. Further transfer of a Glc*N*Ac moiety to lipid I catalyzed by the MurG transferase affords lipid II, which is flipped across the membrane to the periplasm. Then transglycosylation and transpeptidation steps, catalyzed by transglycosylases and transpeptidases, such as penicillin-binding proteins (PBPs), occur to give peptidoglycan, a giant macromolecule that protects the cell against internal osmotic pressure and maintains its shape. Since the enzymes involved in this biosynthesis are ubiquitous and essential among bacteria, they are pertinent targets for developing antibiotics such as fosfomycin-targeting MurA or penicillins and cephalosporins that covalently bind PBPs (Figure 1). However, due to the mechanisms of resistance developed by the bacteria towards these compounds [11,12], it is interesting to focus on other targets, such as the MraY transferase.

**Figure 2:**
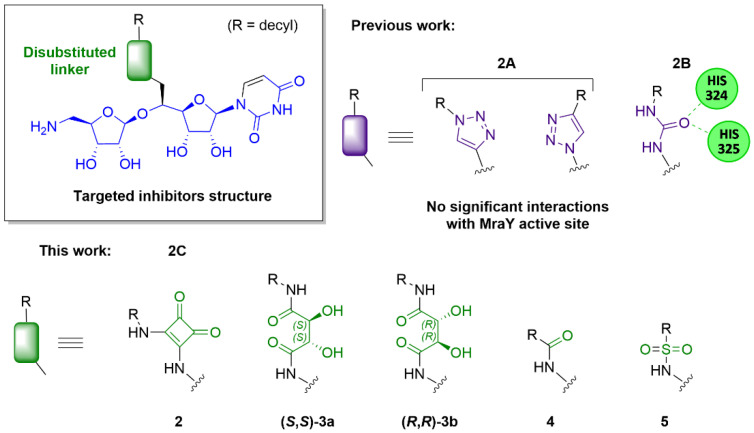
Structure of the synthesized and targeted aminoribosyl uridine-containing inhibitors with various linkers. Previous work: **2A** and **2B**. This work: **2C**.

Several families of potent natural MraY inhibitors are known, such as liposidomycins [13,14,15], muraymycins [16] and caprazamycins [17,18], all of them sharing an aminoribosyluridine moiety as a common structural feature. Currently, there are no MraY-directed antibiotics in clinical use, which is a key advantage to delay the occurrence of resistance. The synthesis of analogs of these peptidonucleosidic compounds has been the matter of intensive synthetic work [19,20,21,22], especially regarding the goal of decreasing their complexity while maintaining potent inhibition of MraY enzymatic activity. This is particularly challenging, considering the hydrophilicity of these molecules, and interesting progress has been achieved towards this objective [23,24,25,26,27]. Based on the aminoribosyluridine skeleton known to be important for MraY inhibition, we developed the synthesis of several families of simplified analogs (Figure 2A), with the chemical diversity being introduced on this scaffold through a triazole [28] or a methylene triazole [29] linker. However, the docking of these triazole-containing inhibitors in either the 5CKR [30] or 6OYH [31] structural models revealed no significant interactions of the triazole with aminoacids of the MraY active site. We then focused on the synthesis of urea-containing compounds [32] with various substituents (Figure 2B), and the most active inhibitor (IC_50_ equal to 1.9 µM) was the one bearing a decyl chain. Molecular dynamics experiments showed that the urea linker could interact with H324 and H325 (Figure 2B), but that this interaction was not stable [32]. This result prompted us to study the effect of other linkers (Figure 2C) that are able to interact by hydrogen bonds with key residues of the MraY active site [31], notably H324 and H325, in order to improve their potency compared to that of the urea-containing compounds. The targeted molecules display various linkers such as squaramide, two isomers of tartaric diamide, amide and sulfonamide (Figure 2C). Indeed, the squaramide ring in compound **2** could act as both a donor and an acceptor of hydrogen bonds [33]. Moreover, the diamide moiety in compounds (*S*,*S*)-**3a** and (*R*,*R*)-**3b** could promote the formation of Supplementary hydrogen bonds due to the presence of two hydroxyl groups [34,35,36]. The amide **4** should permit us to compare the activity of a single amide to that of the diamides (*S*,*S*)-**3a** and (*R*,*R*)-**3b**, whereas the tetrahedral geometry of the sulfur atom and the longer size of the S=O bond in compound **5** could generate hydrogen bonds different from those of the other linkers [37].

An identical C-10 alkyl chain was selected based on the best MraY inhibitory activity of the corresponding urea-containing inhibitor to allow for the accurate comparison of the inhibitory potency among the resulting inhibitors. The docking of these compounds in the structural models 5CKR and 6OYH was performed, and key interactions of the envisaged linkers with D196, N255, N190, L191, D265 and H325 of the MraY active site were highlighted. The stability of the best compounds was confirmed by molecular dynamics experiments. We were delighted to achieve a gain in activity of a factor of 5 with the (*S*,*S*) diamide inhibitor as compared to the urea reference compound, and we wish to present our results in detail.

## 2. Results and Discussion

### 2.1. Chemistry

**Figure 3:**
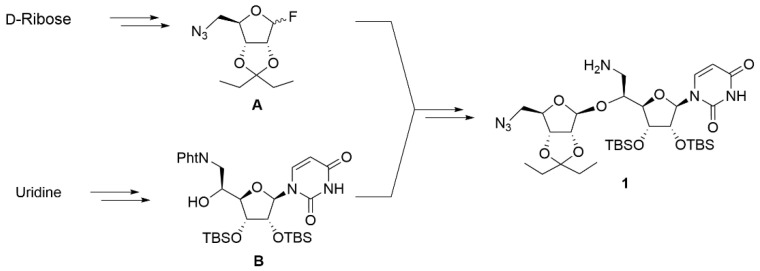
Synthetic route towards the common intermediate **1**.

The targeted compounds were synthesized from the 1″,5″-dideoxy-2″,3″-*O*-isopentylidene-5″-azido-1″-[2′,3′-*O*-isopropylidene-5′(S)-aminomethyl-uridinyl]-β-D-ribofuranose **1** (Figure 3), as we previously described [32]. This common intermediate resulted from the glycosylation of the 5′(*S*)-phthalimidoalcohol **B** [32] with the 5-azidoribosyl fluoride **A** [38] readily obtained in a few steps from uridine and D-ribose, respectively, followed by hydrazinolysis of the phthalimide (Figure 3).

**Scheme 1:**
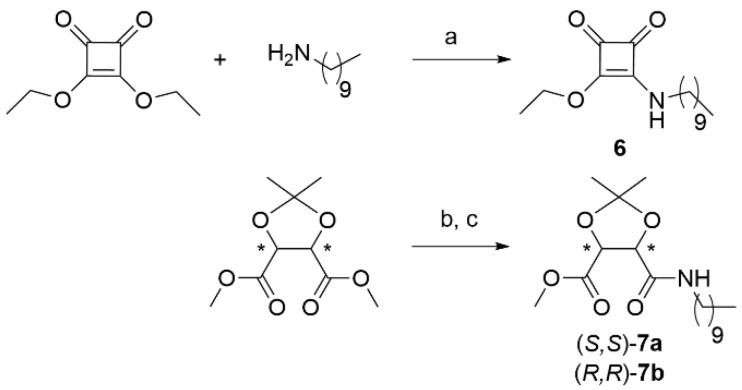
Synthesis of the building blocks **6**, (*S*,*S*)-**7a** and (*R*,*R*)-**7b**. Reagents and conditions: (**a**) TEA, EtOH, r.t., 2 h, 95%; (**b**) KOH, MeOH, r.t., 24 h; (**c**) decylamine, DCC, *N*-hydroxysuccinimide, DMF, r.t., 24 h, 66% over two steps for (*S*,*S*)-**7a**, 60% over 2 steps for (*R*,*R*)-**7b**.

The retrosynthesis towards the targeted compounds is straightforward, the chemical diversity being introduced in the last key step of the synthetic pathway. We first turned to the preparation of the required building blocks. The synthesis of squarate **6** was performed from decylamine and diethyl squarate in ethanol in the presence of triethylamine (Scheme 1). After 2 hours of reaction, the unsymmetrical squarate **6** was isolated in a good 95% yield after purification. For the synthesis of the diamide compounds, both (2*S*,3*S*) and (2*R*,3*R*) isomers of dimethyl tartrate were chosen. Indeed, due to the C_2_ axis of symmetry within these compounds, each of the acid functions is equivalent, and the first reaction on one of these acids, which will desymmetrize tartaric acid, can only lead to the formation of a single diastereomer. Both (2*S*,3*S*) and (2*R*,3*R*) isomers were submitted to a selective monosaponification in the presence of potassium hydroxide in methanol to afford the corresponding acids (Scheme 1). After activation of these intermediates with dicyclohexyl carbodiimide (DCC) and *N*-hydroxysuccinimide (NHS) in DMF for 3 h, peptidic coupling with decylamine for an additional 24 h afforded the crude (*S*,*S*)-**7a** and (*R*,*R*)-**7b**. Flash chromatographic purification of these compounds led to the pure (*S*,*S*)-**7a** compound in 66% yield over two steps and to the pure (*R*,*R*)-**7b** compound in 60% yield over two steps.

**Scheme 2:**
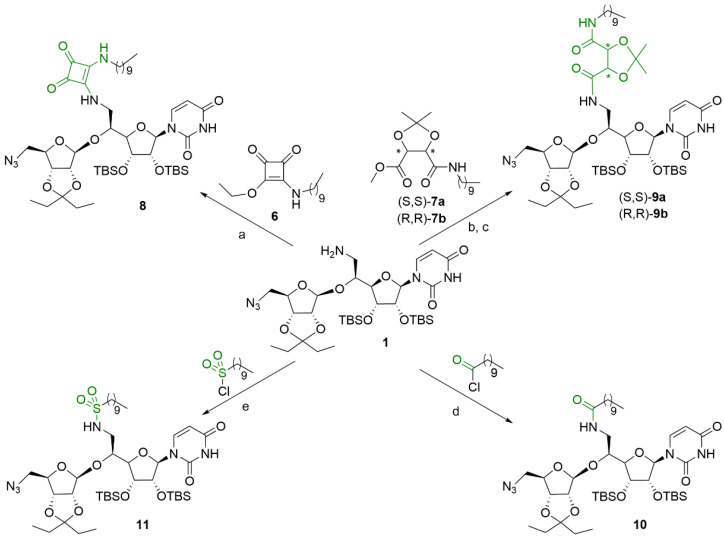
Synthesis of compounds **8–11**. Reagents and conditions: (**a**) **6**, TEA, EtOH, r.t., 30 min, 42%; (**b**) (*S*,*S*)-**7a** or (*R*,*R*)-**7b**, NaOH, MeOH, r.t., 24 h; (**c**) amine **1**, DCC, *N*-hydroxysuccinimide, DMF, r.t., 24 h, 47% for (*S*,*S*)-**9a** and 48% for (*R*,*R*)-**9b** from **1**; (**d**) undecanoylchloride, TEA, DMAP, CH_2_Cl_2_, r.t., 16 h, 72%; (**e**) decanesulfonylchloride, TEA, DMAP, CH_2_Cl_2_, r.t., 16 h, 87%.

With these building blocks in hand, we next turned to the synthesis of compounds **8**–**11** (Scheme 2). The synthesis of the squaramide derivative **8** was carried out by stirring the squarate **6** and the amine **1** in the presence of triethylamine in ethanol for 30 minutes at r.t., which led to the squaramide **8** in 42% yield. The diamide compounds (*S*,*S*)-**9a** and (*R*,*R*)-**9b** resulted from the saponification of the tartrate derivatives (*S*,*S*)-**7a** and (*R*,*R*)-**7b** in the presence of sodium hydroxide in methanol, giving the corresponding acids, followed by their peptidic coupling with the amine **1** that was activated in the presence of DCC and *N*-hydroxysuccinimide in DMF. Compounds (*S*,*S*)-**9a** or (*R*,*R*)-**9b** were isolated in 47 and 48% yield, respectively, from **1**. The condensation of amine **1** with undecanoyl chloride or decanesulfonyl chloride in the presence of triethylamine and 4-dimethylaminopyridine in dichloromethane led to the amide **10** in a satisfactory 72% yield or to the sulfonamide **11** in a good 87% yield.

**Scheme 3:**
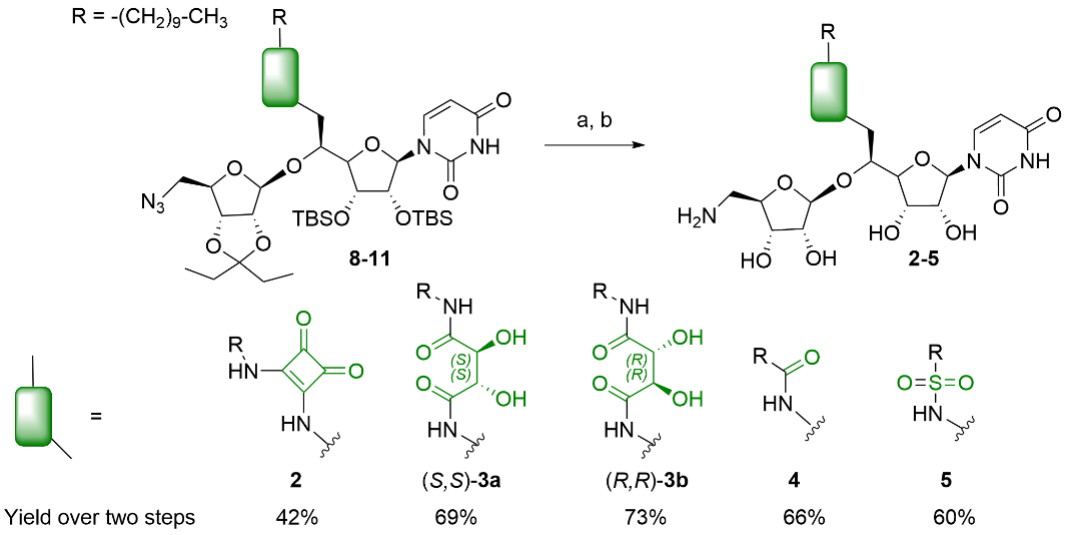
Deprotection steps of compounds **8**–**11**. Reagents and conditions: (**a**) PS-PPh_3_, THF/H_2_O 85/15, r.t., 48 h; (**b**) TFA/H_2_O 4:1, 0 °C, r.t., 16 h.

Finally, the reduction of compounds **8**–**11** was performed in an 85/15 THF/H_2_O mixture under Staudinger conditions, using polymer-supported triphenylphosphine to optimize the removal of supported triphenylphosphine oxide by filtration through a celite pad. Then the acidic hydrolysis of the alcohol protective groups was carried out in a cold 4/1 mixture of trifluoroacetic acid/water. The targeted compounds **2**, (*S*,*S*)-**3a**, (*R*,*R*)-**3b**, **4** and **5** were isolated as their free amine in 42 to 73% yield after flash chromatographic purification on silica gel (Scheme 3).

### 2.2. Biological Studies

**Figure 4:**
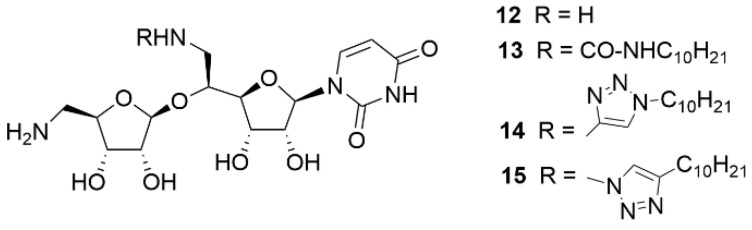
Structure of the amino precursor **12**, urea-, *C*- and *N*-triazole-containing inhibitors **13**, **14** and **15** that we previously synthesized.

The inhibitory activity of the synthesized compounds **2**, (*S*,*S*)-**3a**, (*R*,*R*)-**3b**, **4** and **5** was evaluated on MraY transferase purified from *Aquifex aeolicu*s (MraY_AA_), which was prepared as previously described by Chung et al. [39]. Their activity was compared to the inhibitory activity of the unprotected amino precursor **12**, urea-containing inhibitor **13** and *C*- and *N*-triazole-containing inhibitors **14** and **15** (Figure 4) that we previously synthesized [29,32] (Table 1). Commercially available tunicamycin from *Streptomyces* sp. was used as a positive control in the test.

As shown in Table 1, the five tested compounds are relevant inhibitors of the enzymatic activity catalyzed by the transferase MraY_AA_, with IC_50_ ranging from 0.37 to 17.97 µM. All the synthesized compounds revealed better activity than that of the reference amine **12**, which displayed an IC_50_ value around 50 µM, confirming that substitution of the amine at C6′ is beneficial to the inhibitory effect. Among the tested compounds, the (*S*,*S*)-**3a** diamide displays the strongest inhibition with an IC_50_ equal to 0.37 µM, representing a gain in activity of a factor of **5** as compared to the reference compound **13** with an urea linker. It is noteworthy that its diastereoisomer (*R*,*R*)-**3b** is less active with an IC_50_ equal to 1.38 µM. Interestingly, the simple amide **4** displays an IC_50_ equal to 6.49 µM, thus demonstrating that the presence of the hydroxyl groups in (*S*,*S*)-**3a** and (*R*,*R*)-**3b**, which are capable of creating additional hydrogen bonds with amino acids of the MraY catalytic site, plays a prominent role in enhancing the inhibitory potency of the corresponding inhibitors. The sulfonamide **5** retains good inhibitory activity in the same range as *C*-triazole **14** but is a bit less active than the urea **13** or the *C*-triazole **15**, while the squaramide **2** is probably too rigid for an optimal positioning in the MraY active site, resulting in a significant loss of activity (IC_50_ equal to 17.97 µM). 

Even if our goal was to improve the MraY inhibitory activity of the reference urea **13**, and that was successfully achieved, the antibacterial activity of MraY inhibitors **2**–**5** was also evaluated against several bacterial strains. Gram-negative (*E. coli* ATCC 8730, *C. freundii* ATCC8090 and *P. aeruginosa* ATCC 27853) and Gram-positive pathogenic bacterial strains (*S. aureus* ATCC 25923 and *E. faecium* ATCC 19434), including a methicillin resistant strain (*S. aureus* MRSA ATCC 43300), were selected as representative of pathogen bacterial diversity. Piperacillin and vancomycin were used as positive controls in the tests. As we observed for the urea **13** [32], and as one could expect, none of the tested MraY inhibitors showed antibacterial activity (MICs values were higher than or equal to 128 µg/mL (see Supplementary Materials Table S1) against the six selected bacterial species. Indeed, we previously reported that the urea **13** with a C10 alkyl chain does not display antibacterial activity, since it is probably not able to cross the cytoplasmic membrane to reach its target. Our previous data on differently substituted urea [32] suggested that a linear chain of at least 12 carbon atoms, or a branched substituent, is required to show antibacterial activity. Therefore, further optimization of the lipophilic side chain is still required to increase the antibacterial activity by modulating the lipophilicity of the molecule.

### 2.3. Docking Studies

To predict the binding mode of this series of compounds, we performed docking studies starting from the X-ray crystal structures of MraY_AA_ in complex with muraymycin D2 (MurD2, PDB: 5CKR) [30] and carbacaprazamycin (PDB: 6OYH) [31], using the CDOCKER docking program [40] implemented in the Biovia Discovery Studio docking package 2016. Preferred poses were selected based on the docking score (-CDOCKER interaction energy) and favorable ligand/protein interactions. 

#### 2.3.1. Docking Score

CDOCKER interaction energy included the energy of non-bonded interaction between the protein and the ligand [40]. Higher negative energy values for the docking score indicated stronger binding between MraY_AA_ and the compounds. Tartaric diamides (*S*,*S*)-**3a**, (*R*,*R*)-**3b** performed well in terms of docking scores in comparison to known inhibitors (MurD2, carbacaprazamycin) and urea **13**, both in 5CKR and 6OYH. Lower scores were obtained for hits **2**, **4** and **5** (Table 2). 

#### 2.3.2. Binding Mode of the Hits

Hits bind to hotspots (HSs) of MraY that were identified by Chung et al. [31] by following two modes (one in 5CKR and one in 6OYH), as previously observed for the urea **13** [32].

**Figure 5:**
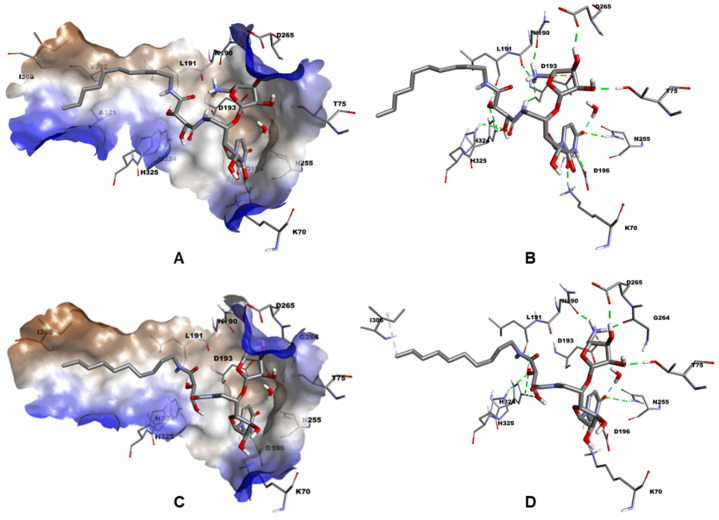
Predicted binding mode of compounds to MraY_AA_ from docking in model 5CKR. (**A**) 3D representation of compound (*S*,*S*)-**3a** with hydrophobic surface of MraY_AA_ rendered as brown and hydrophilic surface as blue. (**B**) 3D diagram of interactions for compound (*S*,*S*)-**3a**. The amino- and diamide-linker moieties of (*S*,*S*)-**3a** engaged an extensive network of H-bond with the residues N190, L191 and D193. (**C**) 3D model for compound (*R*,*R*)-**3b**. (**D**) 2D diagram of interactions for compound (*R*,*R*)-**3b**. Ligands and residues are shown in stick mode. Non-covalent bond interactions are indicated by colored dashed lines: conventional hydrogen bond (green), electrostatic interactions (orange) and water hydrogen bond (blue). The hydrogen atoms of apolar groups were omitted for clarity.

- Binding mode in 5CKR:

The uracil moiety of all compounds occupied the uridine pocket establishing H-bond interactions with K70, N255 and D196 residues (Figure 5). A strong H-bond interaction (d1 < 2.1 Å) involving the key D196 residue [30,42] was retrieved for all compounds, as observed with the urea **13** (Supplementary Materials Table S2) and MurD2 in the crystallographic structure 5CKR (d ~ 1.5 Å). All compounds, except for **4**, formed additional H-bond interactions with both residues K70 and N255, as suggested by distance measurements d2 and d3 (Supplementary Materials Table S2). 

Subtle variations in the binding pattern were observed for the 5-aminoribosyl and linker moieties of new compounds (Table 3). Amide **4** binds to three hotspots (HS1, HS3 and HS5), as previously seen for the urea **13** through the residues K121, N190, D193 and D265. In addition to binding HS1 and HS5, squaramide **2** formed H-bond interactions with H324 and H325 in HS2, while sulfonamide **5** targeted HS1, HS2 and HS3. Focusing on diamide compounds (*S*,*S*)-**3a**, (*R*,*R*)-**3b**, we see that the results analysis revealed that both diastereoisomers have an interesting binding profile combining four hotspots (HS1, HS2, HS5 and HS6, Table 3; see Figure 5). Our binding mode analysis suggests that the extensive network of interactions formed by diamide (*S*,*S*)-**3a** within the MraY binding site may stabilize this compound in a more active conformation. Interestingly, the moderately active compound **2** adopts a strained conformation with spatial reorientation of the aminoribose moiety to minimize steric clashes with H324 and H325, leading to the loss of stabilizing interactions. Taken together, these results suggest that the structural geometry of the linker might guide the positioning of the hit in the binding site. In addition, all compounds oriented their aliphatic tail in the HS2 area, including hydrophobic residues V302, I306 and A321, as seen for the urea **13** in 5CKR model.

**Figure 6:**
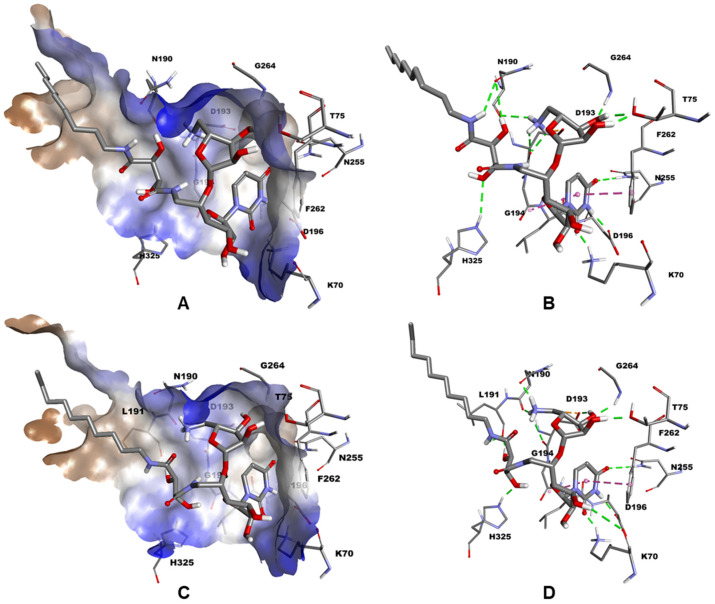
Predicted binding mode of compounds to MraY_AA_ from docking in model 6OYH. Three-dimensional representation (**A**) and 2D diagram of interactions (**B**) for compound (*S*,*S*)-**3a**. Three-dimensional model (**C**) and 2D diagram of interactions (**D**) for compound (*R*,*R*)-**3b**. Ligands and residues are shown in stick mode. Interactions are indicated by colored dashed lines: conventional hydrogen bond (green), electrostatic interactions (orange), and π–π and amide–π interactions (magenta). The hydrogen atoms of apolar groups were omitted for clarity.

- Binding mode in 6OYH:

New hits adopted a second binding mode similar to that observed for our reference compound **13** and carbacaprazamycin in 6OYH model (Figure 6). Compared to the 5CKR model, the major difference is the aliphatic tail shifting from HS2 binding site to the hydrophobic groove (HS4). The anchoring of the uracil moiety involved the same residues, namely K70, D196 and N255, as seen in 5CKR. The strong H-bonding interaction with the D196 residue (d1 ≤ 2Å; see Supplementary Materials Table S2) was preserved for all compounds, and contact with N255 was maintained (d2 ≤ 2.5Å; see Supplementary Materials Table S2). Moreover, a strong H-bond with K70 (d3 ≤ 1.8Å) was also predicted for diamides (*S*,*S*)-**3a** and (*R*,*R*)-**3b**. All ligands, except for **5**, were also stabilized by hydrophobic interactions with residues F262 and G194 (Supplementary Materials data). Furthermore, an in-depth analysis of the binding profile for the aminoribosyl and linker moieties revealed that hits only have one common HS (HS1, Table 4). In addition to HS1, compound **2** bound HS2 and HS3, while compound **4** interacted with HS5 and HS6. More interestingly, interaction with H325 in HS2 was retrieved for both tartrates (*S*,*S*)-**3a** and (*R*,*R*)-**3b**, as previously observed in 5CKR (Table 4). These results suggest that residues D196, N255, K70 (uridine pocket), T75, N190, L191, D193, G264 (HS1), H325 (HS2) and D265 (HS5) may play a key role in ligand binding.

### 2.4. Molecular Dynamics

**Figure 7:**
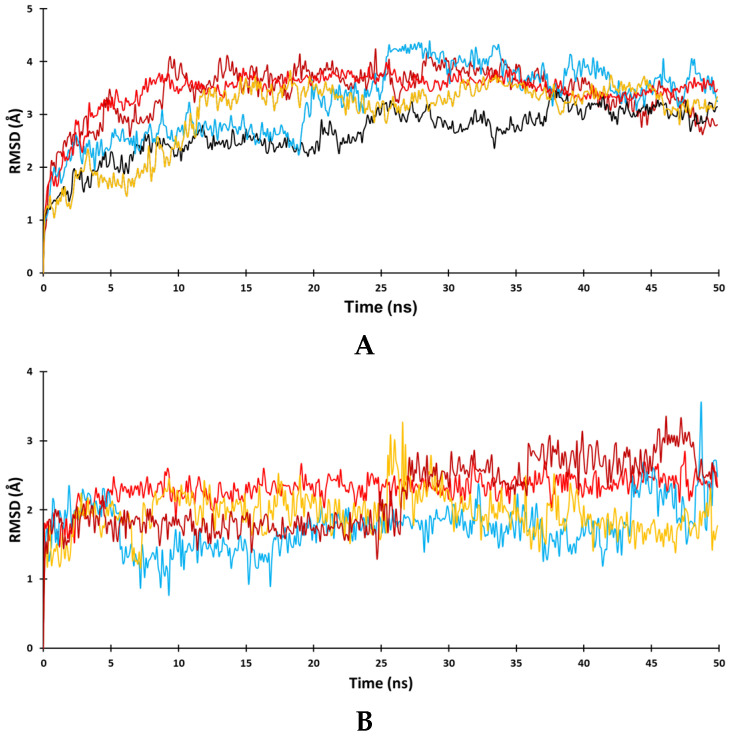
MD simulations of hits targeting the MraY_AA_ binding site. (**A**) RMSD measured on protein backbone atoms for apo MraY_AA_ (black), MraY_AA_/carbacaprazamycin complex (red), MraY_AA_/**13** complex (blue), MraY_AA_/(*S*,*S*)-**3a** complex (orange) and MraY_AA_/(*R*,*R*)-**3b** complex (brown). (**B**) RMSD measured on ligands atoms for carbacaprazamycin (red), **13** (blue), (*S*,*S*)-**3a** (orange) and (*R*,*R*)-**3b** (brown).

To provide insights on the stability of tartaric diamides (*S*,*S*)-**3a** and (*R*,*R*)-**3b** in the binding site of MraY_AA_, we conducted Molecular Dynamics (MD) simulations using NAMD with the CHARMM36m force field [43] implemented in Biovia DS 2021. The experiments were performed starting from the best docking solutions of ligands obtained from CDOCKER and using the procedure previously reported for compound **13** and carbacaprazamycin. Root Mean Standard Deviation (RMSD) analysis of MraY_AA_ and MraY_AA_/ligand complexes measured over protein backbone atoms revealed that all systems are relatively stable along the 50 ns MD runs with no major structural variations (Figure 7A). Diastereoisomer (*S*,*S*)-**3a** displayed a stable RMSD plot similar to that observed for carbacaprazamycin with marginal elevation between 25–30 ns indicating that the ligand binding mode was preserved during the timescale of the simulation (Figure 7B). Diastereoisomer (*R*,*R*)-**3b** seems to be less stable as suggested by a significant elevation of 0.9 Å between 25–50 ns in the deviation profile (Figure 7B).

**Figure 8:**
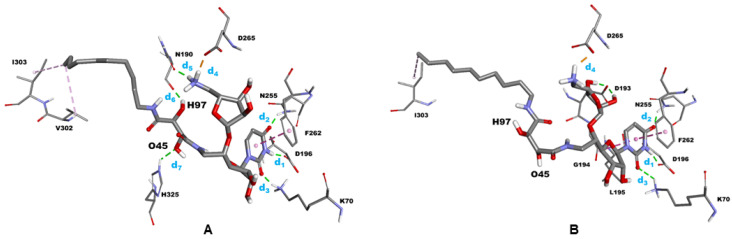
Ligand-binding mode from MD simulations in model 6OYH: (**A**) for compound (*S*,*S*)-**3a** and (**B**) for compound (*R*,*R*)-**3b**. Significant distances measurement (d1–d7) are indicated. For 2D interactions, see Supplementary Materials Figure S4.

MD simulations confirmed the stability of the uracil group for both tartaric diamides (*S*,*S*)-**3a** and (*R*,*R*)-**3b** within the uracil binding pocket through strong H-bond interaction with the residue D196 (d1 ~ 1.8 Å; see Figure 7 and Figure 8A) as seen for the urea compound **13** and carbacaprazamycin (d1 ~ 1.8 Å). The H-bond interaction with N255 was also preserved for both compounds (d2 ~ 2.0 Å; see Supplementary Materials Figure S3A). Interestingly, diamides (*S*,*S*)-**3a** formed an additional H-bond interaction with the residue K70 (d3 ~ 2.7 Å; see Supplementary Materials Figure S3B) suggesting better uracil stability of this compound than the one observed for (*R*,*R*)-**3b**. In addition, the residues F262 and G194 contributed to the binding through hydrophobic π–π interactions (data not shown).

**Figure 9:**
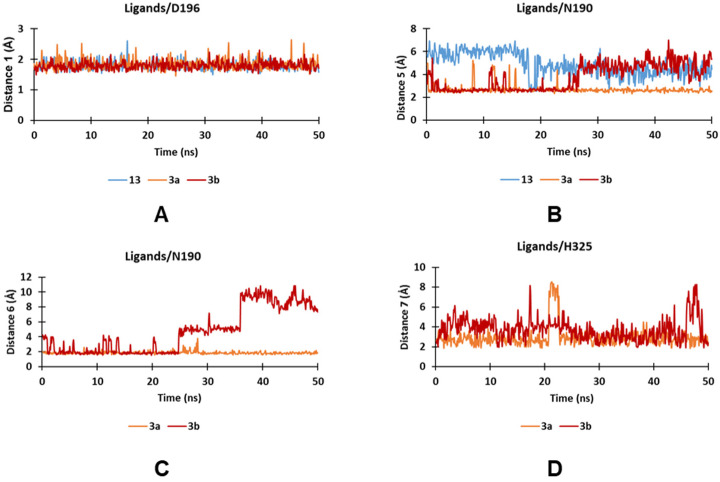
Time evolution of the most significant distances between binding-site residues of MraY_AA_ and ligands atoms. (**A**) Distance d1 between the OD1 atom of D196 and the uridine NH moiety of ligands. (**B**) Distance d5 between the OD1 atom of N190 and the centroid of the amino group of ligands. (**C**) Distance d6 between the O atom of N190 and the H97 atom of the tartrate ligands. (**D**) Distance d7 between the HE1 atom of H325 and the O45 atom of the diamide ligands. (For the other distance measurements, see Supplementary Materials Figure S3).

More variations in the distance profile were retrieved for the aminoribose and linker moieties of both tartaric diamides between 25 and 50 ns. Compound (*S*,*S*)-**3a** established an electrostatic interaction with D265 (d4 ≤ 3 Å for 26% of conformations; see Supplementary Materials Figure S3C). Persistent H-bond interactions were also retrieved with N190 through the amino group (d5 ~ 2.6 Å; see Figure 9B) and the H97 hydrogen atom of the linker (d6 ~ 1.9 Å; see Figure 9C). In addition, the O45 atom of the tartaric moiety formed a stable H-bond with H325 (d7 ~ 2.8 Å; see Figure 9D). Compared to (*S*,*S*)-**3a**, (*R*,*R*)-**3b** lost H-bond interactions with N190 after 25 ns (d6 > 4 Å; see Figure 9C) and H325 contributed weaker to ligand binding (d7 ~ 3.4 Å, Figure 9D) than was observed for the urea linker (d ~ 3.0 Å; data not shown). Interestingly, the position of aliphatic chain for both tartaric diamides remained stable in the hydrophobic groove (HS4) (Figure 8) establishing the same hydrophobic interactions with V302 and I303 as seen for carbacaprazamycin (data not shown), while previous results have revealed that alkyl chain of urea **13** was flipped from HS4 to HS2 after 50 ns MD simulations. Altogether, these results confirmed that (*S*,*S*)-**3a** adopts a more stable conformation than the (*R*,*R*)-**3b** diastereoisomer within the MraY binding site.

## 3. Materials and Methods

### 3.1. Chemical Synthesis

When needed, reactions were carried out under an argon atmosphere. They were monitored by thin-layer chromatography with precoated silica on aluminum foil. Flash chromatography was performed with silica gel 60 (40–63 μm); the solvent systems are given in *v*/*v*. Spectroscopic ^1^H and ^13^C NMR, MS and/or analytical data were obtained by using chromatographically homogeneous samples. ^1^H NMR (500 MHz) and ^13^C NMR (125 MHz) spectra were recorded in CDCl_3_ unless otherwise indicated. Chemical shifts (δ) are reported in ppm, and coupling constants are given in Hz. For each compound, detailed peak assignments were made according to COSY, HSQC and HMBC experiments. The numbering of molecules is indicated in the Supplementary Materials. Optical rotations were measured with a sodium (589 nm) lamp at 20 °C. IR spectra were recorded on an FTIR spectrophotometer, and the wavelengths are reported in cm^−1^. High-resolution mass spectra (HRMS) were recorded with a TOF mass analyzer under electrospray ionization (ESI) in positive ionization mode detection, atmospheric pressure chemical ionization or atmospheric pressure photoionization (APPI).

#### 3.1.1. 3-(Decylamino)-4-ethoxycyclobut-3-ene-1,2-dione **6**

To a solution of diethylsquarate (200 µL, 1.35 mmol, 1 equiv.) in dry EtOH (12 mL) was added triethylamine (565 µL, 4.05 mmol, 3 equiv.) at 0 °C. Decylamine (270 µL, 1.35 mmol, 1 equiv.) was added dropwise to the reaction mixture at 0 °C. The reaction mixture was stirred at r.t. for 2 h, and then solvents were removed in vacuo. Flash chromatography of the residue (pure DCM) afforded **6** as a white solid (364 mg, 95% yield): R*_f_* = 0.75 (DCM/MeOH = 95/5); IR (film) 3261, 2926, 2854, 2250, 1804, 1706, 1611, 1525, 1492, 1457, 1414, 1384, 1338, 1249, 1093, 1056, 916, 867, 732; ^1^H NMR δ 4.78 (q, *J*_H5-H6_ = 7.0 Hz, 2 H, H_5_), 3.42 (dd, *J*_H1′-NH_ = 13.5, *J*_H1′-H2′_ = 6.7 Hz, 2 H, H_1′_), 1.64–1.56 (m, 2 H, H_2′_), 1.46 (t, *J*_H6-H5_ = 7.0 Hz, 3 H, H_6_), 1.34–1.20 (m, 14 H, H_3′_-H_9′_), 0.87 (t, *J*_H10′-H9′_ = 6.9 Hz, 3 H, H_10′_); ^13^C NMR δ 189.7 (C_2_), 182.7 (C_1_), 177.5 (C_4_), 172.5 (C_2_), 77.3 (C_5_), 69.7 (C_1′_), 45.0 (C_2′_), 31.9, 30.6, 29.6, 29.3, 29.2, 26.4, 22.7 (C_3′_-C_9′_), 15.9 (C_6_), 14.2 (C_10′_); HRMS (TOF MS ES^+^) calcd for C_16_H_28_NO_3_^+^ (M + H)^+^ 282.2064, found 282.20609.

#### 3.1.2. Methyl (*2S,3S*)-2,3-O-Isopropylidene-4-(decylamino)-2,3-dihydroxy-4-oxobutanoate (*S*,*S*)-**7a**

KOH (59 mg, 1.04 mmol, 1 equiv.) was dissolved in MeOH (1.5 mL) and added dropwise to a solution of *S*,*S*-protected tartaric acid (228 mg, 1.04 mmol, 1 equiv.) in MeOH (3 mL). The reaction mixture was stirred at 25 °C for 16 h, and then solvents were removed in vacuo. The resulting crude oil was then dissolved in water (15 mL) and washed 3 times with DCM (3 × 15 mL). The aqueous phase was then acidified with 1M HCl until pH 2–3. After evaporation of the water, the compound was dissolved in DCM, and the remaining salts were filtered out. Solvents were removed in vacuo, and the resulting acid was engaged in the next step without further purification. The acid (167 mg, 818 µmol, 1 equiv.) was dissolved in dry DMF (7 mL) under argon. DCC (219 mg, 1.06 mmol, 1.3 equiv.) and *N*-hydroxysuccinimide (99 mg, 858 µmol, 1.05 equiv.) were successively added, and the reaction mixture was stirred at r.t. for 3 h. Decylamine (171 µL, 858 µmol, 105 equiv.) was then added at r.t. After 24 h, solvents were removed in vacuo, and the crude mixture was dissolved in EtOAc and filtered through a celite pad. After removal of the solvent, flash chromatography of the residue (Cyclohexane/EtOAc = 95/5 to 8/2) afforded (*S*,*S*)-**7a** as a pale yellow oil (237 mg, 66% yield over two steps): R*_f_* 0.4 (Cyclohexane/EtOAc = 7/3); [α]_D_ +13.5 (*c* 1.0, MeOH); ^1^H NMR δ 6.51 (broad s, 1 H, NH), 4.74 (s, 2 H, H_2_, H_3_), 3.82 (s, 3 H, CH_3_), 3.31–3.25 (m, 2 H, H_7_), 1.56–1.45 (m, 2 H, H_8_), 1.49 (s, 3 H, H_6a_), 1.48 (s, 3 H, H_6b_), 1.35–1.20 (m, 14 H), 0.87 (t, *J*_H16-H15_ = 6.9 Hz, 3 H, H_16_); ^13^C NMR (126 MHz, CDCl_3_) δ 170.7 (C_4_), 169.4 (C_1_), 113.4 (C_5_), 77.9 (C_3_), 77.6 (C_2_), 53.0 (OCH_3_), 39.3 (C_7_), 32.0 (C_8_), 29.6, 29.4, 29.3, 26.9, 26.8, 26.4 (C_6_), 22.8 (C_15_), 14.2 (C_16_); HRMS (TOF MS ES^+^) calcd for C_18_H_34_NO_5_^+^ (M + H)^+^ 344.2431 found 344.24274.

#### 3.1.3. Methyl (*2R,3R*)-2,3-O-Isopropylidene-4-(decylamino)-2,3-dihydroxy-4-oxobutanoate (*R*,*R*)-**7b**

KOH (57 mg, 1.02 mmol, 1 equiv.) was dissolved in MeOH (1.5 mL) and added dropwise to a solution of *R*,*R*-protected tartaric acid (224 mg, 1.02 mmol, 1 equiv.) in MeOH (3 mL). The reaction mixture was stirred at 25 °C for 16 h, and then solvents were removed in vacuo. The resulting crude oil was then dissolved in water (15 mL) and washed 3 times with DCM (3 × 15 mL). The aqueous phase was then acidified with 1M HCl until pH 2–3. After evaporation of the water, the compound was dissolved in DCM, and the remaining salts were filtered out. Solvent was removed in vacuo, and the resulting acid was engaged in the next step without further purification. The acid (185 mg, 906 µmol, 1 equiv.) was dissolved in dry DMF (7 mL) under argon. DCC (243 mg, 1.18 mmol, 1.3 equiv.) and *N*-hydroxysuccinimide (109 mg, 951 µmol, 1.05 equiv.) were successively added, and the reaction mixture was stirred at r.t. for 3 h. Decylamine (190 µL, 951 mmol, 1.05 equiv.) was then added at r.t. After 24 h, solvents were removed in vacuo, and the crude mixture was dissolved in EtOAc and filtered through a celite pad. After removal of the solvent, flash chromatography of the residue (Cyclohexane/EtOAc = 95/5 to 8/2) afforded (*R*,*R*)-**7b** as a pale yellow oil (210 mg, 60% yield over two steps): R*_f_* 0.4 (Cyclohexane/EtOAc = 7/3); [α]_D_ -16.4 (*c* 1.0, MeOH); ^1^H NMR δ 6.51 (s, 1 H, NH), 4.74 (s, 2 H, H_2_, H_3_), 3.83 (s, 3 H, CH_3_), 3.31–3.26 (m, 2 H, H_7_), 1.56–1.45 (m, 8 H, H_8_ H_6_), 1.34–1.20 (m, 14 H), 0.87 (t, *J*_H16-H15_ = 6.8 Hz, 3 H, H_16_); ^13^C NMR (126 MHz, CDCl_3_) δ 1708 (C_4_), 169.4 (C_1_), 113.4 (C_5_), 77.9 (C_3_), 77.6 (C_2_), 53.0 (OCH_3_), 39.3 (C_7_), 32.0 (C_8_), 29.6, 29.4, 29.3, 27.0, 26.8, 26.4 (C_6_), 22.8 (C_15_), 14.2 (C_16_); HRMS (TOF MS ES^+^) calcd for C_18_H_34_NO_5_^+^ (M + H)^+^ 344.2431 found 344.24271.

#### 3.1.4. Squaramide **8**

To a solution of **6** (13 mg, 42 µmol, 1 equiv.) in dry EtOH (1 mL) was added triethylamine (23 µL, 168 µmol, 4 equiv.) at 0 °C. Amine **1** (37 mg, 50 µmol, 1.2 equiv.) was dissolved in EtOH (1 mL) and added dropwise to the reaction mixture at 0 °C. The reaction mixture was stirred at r.t. for 16 h, and then solvents were removed in vacuo. Flash chromatography of the residue (Cyclohexane/EtOAc = 7/3) afforded squaramide **8** as a colorless oil (16 mg, 35% yield): R*_f_* 0.5 (Cyclohexane/EtOAc = 6/4); [α]_D_ -9 (*c* 1.0, CH_2_Cl_2_); IR (film): 3219, 2928, 2856, 2859, 2370, 2105, 1799, 1695, 1595, 1463, 1263, 1166, 1099, 838, 778; ^1^H NMR δ 7.66 (d, *J*_H6-H5_ = 8.2 Hz, 1 H, H_6_), 6.51 (s, 1 H, NH), 6.00 (s, 1 H, NH), 5.72 (d, *J*_H5-H6_ = 8.2 Hz, 1 H, H_5_), 5.68 (d, *J*_H1′-H2′_ = 4.9 Hz, 1 H, H_1′_), 5.24 (s, 1 H, H_1″_), 4.61 (d, *J*_H3″-H2″_ = 6.4 Hz, 1 H, H_3″_), 4.57 (d, *J*_H2″-H3″_ = 6.4 Hz, 1 H, H_2″_), 4.43–4.35 (m, 1 H, H_4″_), 4.35–4.30 (m, 1 H, H_2′_), 4.17–4.12 (m, 1 H, H_4′_), 4.11–4.05 (m, 1 H, H_3′_), 3.91 (s, 1 H, H_5′_), 3.77–3.69 (m, 1 H, H_6′a_), 3.63–3.56 (m, 3 H, H_1×_ H_5″a_), 3.48 (dd, *J*_H5″b-H5″a_ = 12.9, *J*_H5″b-H4″_ = 7.5 Hz, 1 H, H_5″b_), 3.44–3.40 (m, 1 H, H_6′b_) 1.71 (q, *J*_H7″a-H8″_ = 7.3 Hz, 2 H, H_7″a_), 1.60 (m, 2 H, H_2×_), 1.56 (q, *J*_H7″b-H8″_ = 7.6 Hz, 2 H, H_7″b_), 1.38–1.22 (m, 18 H, -C(CH_3_)_3_), 0.99–0.78 (m, 15 H, SiCH_3_, H_10×_), 0.09 (m, 6 H, H_8″_); ^13^C NMR δ 183.8 (C_9′_), 183.1 (C_10′_), 168.4 (C_8′_), 167.5 (C_11_), 162.7 (C_4_), 150.2 (C_2_), 141.1 (C_6_), 118.4 (C_6″_), 112.1 (C_1″_), 102.2 (C_5_), 90.7 (C_1′_), 85.8 (C_2″_), 85.2 (C_4′_), 84.9 (C_4′_), 81.6 (C_3″_), 81.0 (C_5′_), 74.4 (C_2′_), 71.9 (C_3′_), 53.9 (C_5″_), 45.0 (C_6′_), 44.8 (C_1×_), 32.0, 31.4 (C_2×_), 29.7 (C_7″a_), 29.6 (C_7″b_), 29.5, 29.4, 29.3, 29.3, 29.2, 28.9, 26.5, 26.4, 25.9, 25.7, 22.7, 18.1, 18.0, 14.2, 8.4, 7.6, 0.1, −4.0, −4.5, −4.5, −4.6; HRMS (TOF MS ES^+^) calcd for C_46_H_79_N_7_O_11_Si_2_^+^ (M + H)^+^ 962.5449, found 938.5455.

#### 3.1.5. (*S,S*)-Diamide **9a**

NaOH (7.22 mg, 180 µmol, 1 equiv.) was dissolved in MeOH (0.5 mL) and added dropwise to a solution of (*S*,*S*)-**7a** (62 mg, 180 µmol, 1 equiv.) in MeOH (1 mL). The reaction mixture was stirred at 25 °C for 24 h, and then solvents were removed in vacuo. The resulting crude oil was then dissolved in water (10 mL) and DCM (10 mL). The medium was then acidified with 1M HCl until pH 2–3. Aqueous phase was then extracted three times with DCM. Combined organic phases were washed with brine, dried over Na_2_SO_4_, filtered and concentrated in vacuo. The resulting acid was engaged in the next step without further purification. The resulting acid (23 mg, 69 µmol, 1 equiv.) was dissolved in dry DMF (525 µL) under argon. DCC (18.45 mg, 89 µmol, 1.3 equiv.) and *N*-hydroxysuccinimide (8.31 mg, 72.2 µmol, 1.05 equiv.) were successively added to the reaction mixture and stirred at r.t. for 3 h. Amine **1** (50 mg, 69 µmol, 1 equiv.) was then added at r.t. After 48 h, solvents were removed in vacuo, and the crude mixture was dissolved in EtOAc and filtered through a celite pad. After removal of the solvent, flash chromatography of the residue (Cyclohexane/EtOAc = 6/4) afforded (*S*,*S*)-diamide **9a** as a colorless oil (33.7 mg, 47% yield from **1**): R*_f_* 0.55 (Cyclohexane/EtOAc = 5/5); [α]_D_ -29 (*c* 1.0, MeOH); IR (film): 3346, 2929, 2856, 2105, 1690, 1534, 1462, 1378, 1259, 1216, 1165, 1098, 926, 865, 838, 777, 750; ^1^H NMR δ 8.27 (s, 1 H, H_3_), 7.87 (d, *J*_H6-H5_ = 8.2 Hz, 1 H, H_6_), 7.65–7.57 (m, 1 H, NH), 6.85 (t, *J* = 5.7 Hz, 1 H, NH), 5.79 (d, *J*_H1′-H2′_ = 3.9 Hz, 1 H, H_1′_), 5.71 (dd, *J*_H5-H6_ = 8.2 Hz, 1 H, H_5_), 5.20 (d, 1 H, H_1″_), 4.64 (d, *J*_H3″-H2″_ = 6.2 Hz, 1 H, H_3″_), 4.55 (s, 2 H, H_2×_, H_3×_), 4.53 (d, *J*_H2″-H3″_ = 6.2, 1.0 Hz, 1 H, H_2″_), 4.37 (t, *J*_H4″-H5″_ = 5.4 Hz, 1 H, H_4″_), 4.17 (d, *J*_H2′-H1′_ = 3.9 Hz, 1 H, H_2′_), 4.11 (d, *J*_H4′-H5′_ = 5.1 Hz, 1 H, H_4′_), 4.04–3.97 (m, 1 H, H_3′_), 3.91–3.84 (m, 1 H, H_5′_), 3.77–3.68 (m, 1 H, H_6′a_), 3.65–3.55 (m, 1 H, H_6′b_), 3.50 (dd, *J*_H5″a-H5″b_ = 12.9, *J*_H5″-H4″_ = 5.4 Hz, 2 H, H_5″_), 3.28 (t, *J*_H7×-H8×_ = 7.0 Hz, 2 H, H_7×_), 1.71 (q, *J*_H7″a-H8″_ = 7.3 Hz, 2 H, H_7″a_), 1.56 (q, *J*_H7″b-H8″_ = 7.3 Hz, 2 H, H_7″b_), 1.47 (s, 3 H, H_6×a_), 1.46 (s, 3 H, H_6×b_), 1.35–1.22 (m, 18 H), 0.96–0.83 (m, 24 H, -C(CH_3_)_3_, H_8″_), 0.09 (m, 12 H, SiCH_3_); ^13^C NMR δ 170.3 (C_4×_), 169.5 (C_1×_), 162.8 (C_4_), 150.0 (C_2_), 140.2 (C_6_), 118.3 (C_6″_), 112.5 (C_5×_), 112.2 (C_1″_), 101.8 (C_5_), 89.6 (C_1′_), 86.0 (C_2″_), 85.0 (C_4″_), 84.9 (C_4′_), 81.7 (C_3″_), 78.9 (C_5′_), 77.6 (C_3×_), 77.5 (C_2×_), 75.4 (C_2′_), 71.7 (C_3′_), 53.5 (C_5″_), 41.8 (C_6′_), 39.5 (C_7×_), 31.9, 29.6, 29.6, 29.4, 29.3, 29.3, 29.0, 27.0, 26.3, 26.2, 25.9, 25.9, 25.8, 25.8, 22.7, 18.1, 14.2, 8.4, 7.6, −3.9, −4.3, −4.7; HRMS (TOF MS ES^+^) calcd for C_49_H_88_N_7_O_13_Si_2_^+^ (M + H)^+^ 1038.5973 found 1038.59913.

#### 3.1.6. (*R,R*)-Diamide **9b**

NaOH (9.67 mg, 241 µmol, 1 equiv.) was dissolved in MeOH (0.5 mL) and added dropwise to a solution of (*R*,*R)*-**7b** (83 mg, 241 µmol, 1 equiv.) in MeOH (1 mL). The reaction mixture was stirred at 25 °C for 24 h, and then solvents were removed in vacuo. The resulting crude oil was then dissolved in water (10 mL) and DCM (10 mL). The medium was then acidified with 1M HCl until pH 2–3. Aqueous phase was then extracted three times with DCM. Combined organic phases were washed with brine, dried over Na_2_SO_4_, filtered and concentrated in vacuo. The resulting acid was engaged in the next step without further purification. The resulting acid (23 mg, 69 µmol, 1 equiv.) was dissolved in dry DMF (525 µL) under argon. DCC (18.45 mg, 89 µmol, 1.3 equiv.) and *N*-hydroxysuccinimide (8.31 mg, 72.2 µmol, 1.05 equiv.) were successively added, and the reaction mixture was stirred at r.t. for 3 h. Amine **1** (50 mg, 69 µmol, 1 equiv.) was then added at r.t. After 48 h, solvents were removed in vacuo, and the crude mixture was dissolved in EtOAc and filtered through a celite pad. After removal of the solvent, flash chromatography of the residue (Cyclohexane/EtOAc = 6/4) afforded (*R*,*R*)-**9b** as a colorless oil (34.4 mg, 48% yield from **1**): R*_f_* 0.55 (Cyclohexane/EtOAc = 5/5); [α]_D_ +13 (*c* 1.0, MeOH); IR (film): 3005, 2988, 2929, 2857, 2105, 1693, 1541, 1462, 1384, 1275, 1268, 1167, 1098, 925, 871, 839 764, 750; ^1^H NMR δ 8.11 (s, 1 H, H_3_), 7.88 (d, *J*_H6-H5_ = 8.2 Hz, 1 H, H_6_), 7.80 (t, *J*_H4×-H7×_ = 5.0 Hz, 1 H, NH_4×_), 6.85 (t, *J*_H7′-H6′_ = 5.4 Hz, 1 H, NH_7′_), 5.77 (d, *J*_H1′-H2′_ = 3.7 Hz, 1 H, H_1′_), 5.70 (d, *J*_H5-H6_ = 8.2 Hz, 1 H, H_5_), 5.17 (s, 1 H, H_1″_), 4.65 (dd, *J*_H3″-H2″_ = 6.2 Hz, 1 H, H_3″_), 4.53 (m, 2 H, H_2×_, H_2″_), 4.49 (d, *J*_H3×-H2×_ = 7.2 Hz, 1 H, H_3×_), 4.39 (t, *J*_H4″-H5″_ = 5.4 Hz, 1 H, H_4″_), 4.17 (d, *J*_H2′-H1′_ = 3.7 Hz, 1 H, H_2′_), 4.14 (d, *J*_H4′-H3′_ = 5.3 Hz, 1 H, H_4′_), 3.99 (d, *J*_H3′-H4′_ = 5.3 Hz, 1 H, H_3′_), 3.87–3.80 (m, 1H, H_5′_), 3.76 (ddd, *J*_H6′a-H6′b_ = 14.2, *J*_H6′a-H5′_ = 7.2, *J*_H6′a-H7′_ = 5.4 Hz, 1 H, H_6′a_), 3.59–3.50 (m, 3 H, H_5″_, H_6′b_), 3.28 (dd, *J*_H7×-H8×_ = 13.7, *J*_H7×-NH×_ = 5.0 Hz, 1 H, H_7×_), 1.70 (q, *J*_H7″a-H8″_ = 7.3 Hz, 2 H, H_7″a_), 1.56 (q, *J*_H7″b-H8″_ = 7.3 Hz, 2 H, H_7″b_), 1.39 (m, 6 H), 1.37–1.18 (m, 6 H), 0.96–0.81 (m, 24 H, -C(CH_3_)_3_, H_8″_), 0.10 (2s, 12 H, SiCH_3_); ^13^C NMR δ 170.1 (C_4×_), 169.6 (C_1×_), 162.8 (C_4_), 150.0 (C_2_), 140.3 (C_6_), 118.1 (C_6″_), 112.5 (C_1″_), 101.6 (C_5_), 89.7 (C_1′_), 86.04 (C_2″_), 85.1 (C_4″_), 84.9 (C_4′_), 81.84 (C_3″_), 79.5 (C_5′_), 77.7 (C_2×_), 77.5 (C_3×_), 75.4 (C_2′_), 71.7 (C_3′_), 53.5 (C_5″_), 41.77 (C_6′_), 39.4 (C_7×_), 32.00, 29.63, 29.60, 29.40, 29.36, 29.3 (C_7″a_), 28.9 (C_7″b_), 26.9, 26.2, 26.0, 25.9, 25.9 (C_6×_), 25.8, 22.7, 18.1, 14.2, 8.4, 7.5, −3.9, −4.3, −4.7; HRMS (TOF MS ES^+^) calcd for C_49_H_88_N_7_O_13_Si_2_^+^ (M + H)^+^ 1038.5973 found 1038.5987.

#### 3.1.7. Amide **10**

To a solution of **1** (50 mg, 69 µmol, 1 equiv.) in dry DCM (1 mL) were successively added, at 0 °C, triethylamine (19 µL, 138 µmol, 2 equiv.), DMAP (9 mg, 69 µmol, 1 equiv.) and undecanoyl chloride (18 µl, 83 µmol, 1.2 equiv.). The reaction mixture was stirred at r.t. for 12 h, and then solvents were removed in vacuo. Flash chromatography of the residue (Cyclohexane/EtOAc = 7/3) afforded the amide **10** as a colorless oil (44 mg, 72% yield): R*_f_* 0.5 (Cyclohexane/EtOAc = 6/4); [α]_D_ -8 (*c* 1.0, MeOH); IR (film): 2929, 2857, 2106, 1690, 1540, 1463, 1379, 1275, 1260, 1167, 1138, 1099, 927, 887, 840, 764, 750; ^1^H NMR δ 8.81 (s, 1 H, NH), 7.83 (d, *J*_H6-H5_ = 8.2 Hz, 1 H, H_6_), 6.51 (d, *J*_NH7′-H6′_ = 7.4 Hz, 1 H, NH_7′_), 5.74 (d, *J*_H1′-H2′_ = 3.7 Hz, 1 H, H_1′_), 5.70 (d, *J*_H5-H6_ = 8.2, 1 H, H_5_), 5.18 (s, 1 H, H_1″_), 4.61 (d, *J*_H3″-H2″_ = 6.3 Hz, 1 H, H_3″_), 4.52 (d, *J*_H2″-H3″_ = 6.3 Hz, 1 H, H_2″_), 4.40 (t, *J*_H4″-H5″_ = 5.1 Hz, 1 H, H_4″_), 4.19 (d, *J*_H2′-H1′_ = 3.7 Hz, 1 H, H_2′_), 4.11 (d, *J*_H3′-H4′_ = 5.2 Hz, 1 H, H_3′_), 4.02–3.96 (m, 1 H, H_4′_), 3.81 (dd, *J*_H5′-H6′_ = 4.1, *J*_H5′-H4′_ = 2.4 Hz, 1 H, H_5′_), 3.80–3.74 (m, 1 H, H_6′a_), 3.49 (ddd, *J*_H5″a-H5″b_ = 19.7, *J*_H5″-H4″_ = 5.1 Hz, 2 H, H_5″_), 3.35 (ddd, *J*_H6′b-H6′a_ = 14.1, *J*_H6′b-NH7′_ = 7.4, *J*_H6′b-H5′_ = 4.1 Hz, 1 H, H_6′b_), 2.19 (t, *J*_H1×-H2×_ = 7.6 Hz, 2 H, H_1×_), 1.70 (q, *J*_H7″a-H8″_ = 7.3 Hz, 2 H, H_7″a_), 1.67–1.59 (m, 2 H, H_2×_), 1.54 (q, *J*_H7″b-H8″_ = 7.3 Hz, 2 H, H_7″b_), 1.27 (m, 16 H), 0.95–0.81 (m, 24 H, -C(CH_3_)_3_, H_8″_), 0.16–0.03 (m, 15 H, SiCH_3_, H_10×_); ^13^C NMR δ 173.5 (C_8′_), 163.2 (C_4_), 150.1 (C_2_), 140.4 (C_6_), 118.2 (C_6″_), 112.2 (C_1″_), 101.7 (C_5_), 90.0 (C_1′_), 86.0 (C_2″_), 85.0 (C_4′_), 84.9 (C_4″_), 81.7 (C_3″_), 80.2 (C_5′_), 75.2 (C_2′_), 71.6 (C_3′_), 53.8 (C_5″_), 41.9 (C_6″_), 36.9 (C_1×_), 32.0 (C_3×_), 29.7 (C_7″a_), 29.6 (C_7″b_), 29.5, 29.4, 29.4, 29.3, 28.9, 25.9, 25.9, 25.8, 25.7, 22.7, 18.1, 14.2, 8.4, 7.6, −3.9, −4.3, −4.7; HRMS (TOF MS ES^+^) calcd for C_43_H_78_N_6_O_10_Si_2_^+^ (M + H)^+^ 895.5391, found 895.5404.

#### 3.1.8. Sulfonamide **11**

To a solution of **1** (45 mg, 62 µmol, 1 equiv.) in dry DCM (4 mL) were successively added, at 0 °C, DMAP (9 mg, 69 µmol, 1.2 equiv.) and undecanoylsulfonyl chloride (18 µL, 74 µmol, 1.2 equiv.). The reaction mixture was stirred at r.t. for 16 h, and then solvents were removed in vacuo. Flash chromatography of the residue (Cyclohexane/EtOAc = 7/3) afforded sulfonamide **11** as a colorless oil (50 mg, 87% yield): R*_f_* 0.3 (Cyclohexane/EtOAc = 7/3); [α]_D_ -19 (*c* 1.0, CH_2_Cl_2_); IR (film): 2929, 2897, 2104, 1632, 1413, 1329, 1275, 1260, 1140, 1101, 926, 840, 764, 750. ^1^H NMR δ 7.85 (d, *J*_H6-H5_ = 8.2 Hz, 1 H, H_6_), 5.75 (d, *J*_H1′-H2′_ = 3.4 Hz, 1 H, H_1′_), 5.72 (d, *J*_H5-H6_ = 8.3 Hz, 1 H, H_5_), 5.60 (d, *J*_NH7′-H6′_ = 7.3 Hz, 1 H, NH_7′_), 5.22 (s, 1 H, H_1″_), 4.63 (d, *J*_H2′-H1′_ = 3.4 Hz, 1 H, H_2′_), 4.53–4.51 (m, 1 H, H_3′_), 4.44–4.41 (m, 1 H, H_4′_), 4.19–4.16 (m, 1 H, H_4″_), 4.15 (d, *J*_H2″-H3″_ = 5.7 Hz, 1 H, H_2″_), 4.01 (d, *J*_H3″-H2″_ = 5.7 Hz, 1 H, H_3″_), 3.91–3.88 (m, 1 H, H_5′_), 3.59 (dd, *J*_H5″a-H5″b_ = 13.0, *J*_H5″a-H4″_ = 4.5 Hz, 1 H, H_5″a_), 3.51–3.46 (m, 1 H, H_5″b_), 3.40 (dd, *J*_H6′-NH7′_ = 7.3, *J*_H6′-H5′_ = 2.5 Hz, 2 H, H_6′_), 3.01 (t, *J*_H1×-H2×_ = 6.8, 2 H, H_1×_), 1.84–1.76 (m, 2 H, H_2×_), 1.70 (q, *J*_H7″a-H8″_ = 7.3 Hz, 2 H, H_7″a_), 1.56 (q, *J*_H7″b-H8″_ = 7.4 Hz, 2 H, H_7″b_), 1.43–1.36 (m, 4 H), 1.27 (m, 24 H, -C(CH_3_)_3_, H_8″_), 1.01–0.67 (m, 12 H), 0.25–0.01 (m, 15 H, SiCH_3_, H_10×_); ^13^C NMR δ 163.4 (C_6_), 150.2 (C_2_), 140.1 (C_6_), 118.2 (C_6″_), 111.9 (C_1″_), 101.7 (C_5_), 89.8 (C_1′_), 85.9 (C_2″_), 85.2 (C_4′_), 84.6 (C_4″_), 81.6 (C_3″_), 80.7 (C_5′_), 75.2 (C_2′_), 71.8 (C_3′_), 53.6 (C_5″_), 53.0 (C_1×_), 45.7 (C_6′_), 31.9, 29.6 (C_7″a_), 29.5, 29.4, 29.3, 29.3, 29.2, 29.0, 28.4 (C_7″b_), 25.9, 25.9, 25.8, 25.8, 25.8, 23.7 (C_2×_), 22.7, 18.0, 18.0, 14.1, 8.4, 7.6, −3.9, −4.2, −4.8; HRMS (TOF MS ES^+^) calcd for C_42_H_79_N_6_O_11_SSi_2_^+^ (M + H)^+^ 931.5066, found 931.5109.

##### General Procedure for Compounds Deprotection

To a solution of the protected compounds **8**–**11** (1 equiv.) in dry THF (2 mL) was added Polymer-supported triphenylphosphine (3 mmol/g; 6 equiv.) and pure water (0.7 mL). The reaction mixture was carefully stirred at r.t. for 48 h. The reaction was then filtered through a celite pad, carefully rinsed with THF and concentrated in vacuo to afford the crude amine. To the crude residue was added pure H_2_O, and the resulting suspension was stirred at 0 °C. Then, at 0 °C, TFA (300 equiv.) was added dropwise. The orange resulting solution was stirred at 0 °C for 10 min and then at r.t. for 18 h. After concentration in vacuo, flash chromatography of the residue (DCM/MeOH/NH4OH 14% 80/18/2) afforded the fully deprotected compounds (**2**–**5**) in 42 to 73% yield over two steps

#### 3.1.9. Squaramide **2**

Compound **2** was prepared according to the general procedure for compounds deprotection from the squaramide **2** (16 mg, 17 μmol, 1 equiv.) and was obtained as a colorless oil (5 mg, 43% yield over 2 steps): R*_f_* 0.25 (DCM/MeOH/NH_4_OH 14% 80/18/2); [α]_D_ + 9 (*c* 1.0, CH_2_Cl_2_); IR (film): 2934, 2927, 2368, 2340, 1756, 1715, 1275, 1260, 764, 750; ^1^H NMR (500 MHz, MeOD) δ 7.81 (d, *J*_H6-H5_ = 8.1 Hz, 1 H, H_6_), 5.81 (s, 1 H, H_1′_), 5.72 (d, *J*_H5-H6_ = 8.1 Hz, 1 H, H_5_), 5.19 (s, 1 H, H_1″_), 4.20–4.13 (m, 2 H, H_2′_, H_3′_), 4.10 (m, 2 H, H_4′_ H_4″_), 4.05 (bs, 1 H, H_2″_), 3.97 (d, *J*_H3″-H2″_ = 4.2 Hz, 1 H, H_3″_), 3.83 (td, *J*_H5′-H6′_ = 11.4, *J*_H5′-H4′_ = 6.3 Hz, 1 H, H_5′_), 3.63 (d, *J*_H6′-H5′_ = 11.4 Hz, 2 H, H_6′_), 3.44 (d, *J*_H5″-H4″_ = 8.0 Hz, 2 H, H_5″_), 3.19–3.10 (m, 2 H, H_1×_), 1.61 (t, *J*_H2×-H1×_ = *J*_H2×-H3×_ = 6.9 Hz, 2 H, H_2×_), 1.44–1.16 (m, 14 H), 0.90 (t, *J*_H10×-H9×_ = 6.8 Hz, 3 H, H_10×_); ^13^C NMR (125 MHz, MeOD) δ 183.9 (C_10′_ C_9′_), 173.0 (C_11′_ C_8′_), 166.1 (C_4_), 152.1 (C_2_), 142.2 (C_6_), 110.0 (C_1″_), 102.6 (C_5_), 92.2 (C_1′_), 84.1 (C_3′_), 79.9 (C_4″_), 76.2 (C_3″_), 75.5 (C_2′_), 73.8 (C_2″_), 70.9 (C_4′_), 45.4 (C_1×_), 44.6 (C_5″_), 35.3 (C_6′_), 33.1 (C_2×_), 32.2, 27.6, 27.4, 24.2, 23.7, 23.3, 20.8 (C_3×_–C_9×_), 14.5 (C_10×_); HRMS (TOF MS ES^+^) calcd for C_29_H_46_N_5_O_11_^+^ (M + H)^+^ 640.3188, found 640.3203.

#### 3.1.10. (*S*,*S*)-Diamide **3a**

Compound **3a** was prepared according to the general procedure for compounds deprotection from diamide **9a** (25 mg, 25 μmol, 1 equiv.) and was obtained as a white powder (11.5 mg, 69% yield over two steps): R*_f_* 0.15 (DCM/MeOH/NH_4_OH 14% 80/18/2); [α]_D_ +19 (*c* 1.0, MeOH); IR (film): 3400, 2925, 1670, 1658, 1466, 1275, 1264, 1123, 764, 750; ^1^H NMR (500 MHz, MeOD) δ 7.83 (d, *J*_H6-H5_ = 8.1 Hz, 1 H, H_6_), 5.82 (d, *J*_H1′-H2′_ = 2.5 Hz, 1 H, H_1′_), 5.72 (d, *J*_H5-H6_ = 8.1 Hz, 1 H, H_5_), 5.12 (s, 1 H, H_1″_), 4.49 (d, *J*_H2×-H3×_ = 1.6 Hz, 1 H, H_2×_), 4.47 (d, *J*_H3×-H2×_ = 1.6 Hz, 1 H, H_3×_), 4.15 (m, 2 H, H_2′_, H_3′_), 4.12–4.04 (m, 2 H, H_4′_, H_4″_), 4.04–3.96 (m, 2 H, H_2″_, H_3″_), 3.71 (td, *J*_H5′-H6′_ = 14.1, *J*_H5′-H4′_ = 4.3 Hz, 1 H, H_5′_), 3.43 (dd, *J*_H6′-H5′_ = 14.1, *J*_H6′-H7′_ = 7.3 Hz, 2 H, H_6′_), 3.28–3.16 (m, 2 H, H_5″_), 3.03 (td, *J*_H7×-H8×_ = 13.3, *J*_H7×-H4×_ = 9.4 Hz, 2 H, H_7×_), 1.57–1.50 (m, 2 H, H_8×_), 1.37–1.24 (m, 14 H), 0.90 (t, *J*_H16×-H15×_ = 6.9 Hz, 3 H, H_16×_); ^13^C NMR (125 MHz, MeOD) δ 174.8 (C_1×_), 174.1 (C_4×_), 166.2 (C_4_), 152.1 (C_2_), 142.1 (C_6_), 111.4 (C_1″_), 102.4 (C_5_), 91.9 (C_1′_), 84.2 (C_3′_), 80.1 (C_4″_), 77.4 (C_5′_), 77.5 (C_2×_), 76.9 (C_3×_), 76.1 (C_3″_), 75.7 (C_2′_), 73.7 (C_2″_), 71.0 (C_4′_), 45.2 (C_5″_), 42.4 (C_6′_), 36.1 (C_7×_), 33.1 (C_8×_), 30.7, 30.7, 30.7, 30.7, 30.5, 26.9, 23.8 (C_9×_–C_15×_), 14.5 (C_16×_); HRMS (TOF MS ES^+^) calcd for C_29_H_50_N_5_O_13_^+^ (M + H)^+^ 676.3400 found 676.34095.

#### 3.1.11. (*R*,*R*)-Diamide **3b**

Compound **3b** was prepared according to the general procedure for compounds deprotection from diamide **9b** (25 mg, 25 μmol, 1 equiv.) and was obtained as a white powder (12.2 mg, 73% yield over two steps): R*_f_* 0.15 (DCM/MeOH/NH_4_OH 14% 80/18/2); [α]_D_ -8 (*c* 1.0, MeOH); IR (film) 3383, 2925, 1657, 1467, 1275, 1268, 1130, 764, 750; ^1^H NMR (500 MHz, MeOD) δ 7.82 (d, *J*_H6-H5_ = 8.1 Hz, 1 H, H_6_), 5.78 (d, *J*_H1′-H2′_ = 2.7 Hz, 1 H, H_1′_), 5.71 (d, *J*_H5-H6_ = 8.1 Hz, 1 H, H_5_), 5.09 (d, *J* = 1.0 Hz, 1 H, H_1″_), 4.53 (d, *J*_H2×-H3×_ = 1.6 Hz, 1 H, H_2×_), 4.51 (d, *J*_H3×-H2×_ = 1.6 Hz, 1 H, H_3×_), 4.17 (dd, *J*_H1′-H1′_ = 2.7 Hz, 1 H, H_2′_), 4.16–4.13 (m, 1 H, H_3′_), 4.07 (d, *J*_H2″-H3″_ = 7.2 Hz, 1 H, H_2″_), 4.06–4.01 (m, 2 H, H_4′_, H_4″_), 4.00–3.95 (m, 1 H, H_3″_), 3.80 (dd, *J*_H5′a-H5′b_ = 14.1, *J*_H5′-H4′_ = 4.0 Hz, 1 H, H_5′_), 3.40–3.33 (m, 2 H, H_6′_), 3.29–3.22 (m, 2 H, H_5″_), 3.21–3.13 (m, 2 H, H_7×_), 1.57–1.51 (m, 2 H, H_8×_), 1.39–1.23 (m, 14 H), 0.90 (t, *J* = 7.0 Hz, 3 H, H_16×_); ^13^C NMR (125 MHz, MeOD) δ 174.8 (C_1×_), 174.1 (C_4×_), 166.2 (C_4_), 152.1 (C_2_), 142.4 (C_6_), 111.0 (C_1″_), 102.4 (C_5_), 92.2 (C_1′_), 84.0 (C_3′_), 80.4 (C_4″_), 77.3 (C_5′_), 77.0 (C_2×_), 76.8 (C_3×_), 76.4 (C_3″_), 75.4 (C_2′_), 74.0 (C_2″_), 71.3 (C_4′_), 44.9 (C_5″_), 42.0 (C_6′_), 36.5 (C_7×_), 33.0 (C_8×_), 30.7, 30.5, 30.4, 30.4, 30.3, 26.9, 23.4 (C_9×_–C_15×_), 14.5 (C_16×_); HRMS (TOF MS ES^+^) calcd for C_29_H_50_N_5_O_13_^+^ (M + H)^+^ 676.3400 found 676.3411.

#### 3.1.12. Amide **4**

Compound **4** was prepared according to the general procedure for compounds deprotection from amide **10** (23 mg, 25 μmol, 1 equiv.) and was obtained as a white powder (42 mg, 66% yield over two steps). R*_f_* 0.15 (DCM/MeOH/NH_4_OH 14% 80/18/2); [α]_D_ +10 (*c* 1.0, MeOH); IR (film): 3749, 2815, 1597, 1541, 1306, 1275, 1260, 1000, 849, 764, 750; ^1^H NMR (500 MHz, MeOD) δ 7.83 (d, *J*_H6-H5_ = 8.1 Hz, 1H, H_6_), 5.81 (d, *J*_H1′-H2′_ = 2.9 Hz, 1H, H_1′_), 5.71 (d, *J*_H5-H6_ = 8.1 Hz, 1H, H_5_), 5.12 (s, 1H, H_1″_), 4.14 (d, *J*_H2′-H1′_ = 2.9 Hz, 1H, H_2′_), 4.13–4.08 (m, 2H, H_3′_, H_4′_), 4.08–4.03 (m, 2H, H_4″_, H_2″_), 3.98 (d, *J*_H3″-H2″_ = 4.5 Hz, 1H, H_3″_), 3.91 (td, *J*_H5′-H6′b_ = 7.9, *J*_H5′-H4′_ = 4.6 Hz, 1H, H_5′_), 3.66 (d, *J*_H6′a-H6′b_ = 13.6 Hz, 1H, H_6′a_), 3.40 (dd, *J*_H6′b-H6′a_ = 13.7, *J*_H66′b-H5′_ = 7.9 Hz, 1H, H_6′b_), 3.35 (m, 1H, H_5″a_), 3.08 (dd, *J*_H5″b-H5″a_ = 13.0, *J*_H5″b-H4″_ = 9.8 Hz, 1 H, H_5″b_), 2.25–2.18 (m, 2 H, H_1×_), 1.64–1.57 (m, 2 H, H_2×_), 1.32 (dd, *J* = 13.4, 8.3 Hz, 14 H), 0.90 (t, *J*_H10×-H9×_ = 7.0 Hz, 3 H, H_10×_); ^13^C NMR (125 MHz, MeOD) δ 176.8 (C_8′_), 166.1 (C_4_), 152.1 (C_2_), 142.1 (C_6_), 110.8 (C_1″_), 102.4 (C_5_), 91.7 (C_1′_), 84.3 (C_3′_), 80.2 (C_4″_), 77.3 (C_5′_), 76.3 (C_3″_), 75.6 (C_2′_), 74.0 (C_2″_), 70.9 (C_4′_), 44.7 (C_5″_), 42.1 (C_6′_), 37.1 (C_1×_), 33.0 (C_2×_), 30.7, 30.6, 30.4, 30.4, 30.3, 26.9, 23.7 (C_3×_–C_9×_), 14.4 (C_10×_); HRMS (TOF MS ES^+^) calcd for C_26_H_45_N_4_O_10_^+^ (M + H)^+^ 573.3130, found 573.3145.

#### 3.1.13. Sulfonamide **5**

Compound **5** was prepared according to the general procedure for compounds’ deprotection from sulfonamide **11** (37 mg, 41 μmol, 1 equiv.) and was obtained as a white powder (15 mg, 60% yield over 2 steps): R*_f_* 0.15 (DCM/MeOH/NH_4_OH 14% 80/18/2); IR (film): 2929, 2105, 1697, 1463, 1275, 1260, 1100, 874, 838, 764, 750; ^1^H NMR (500 MHz, MeOD) δ 7.81 (d, *J*_H6-H5_ = 8.1 Hz, 1 H, H_6_), 5.78 (d, *J*_H1′-H2′_ = 2.8 Hz, 1 H, H_1′_), 5.71 (d, *J*_H5-H6_ = 8.1 Hz, 1 H, H_5_), 5.16 (s, 1 H, H_1″_), 4.17 (d, *J*_H2′-H1′_ = 2.8 Hz, 1 H, H_2′_), 4.15–4.12 (m, 1 H, H_3′_), 4.12–4.09 (m, 2 H, H_4′_, H_4″_), 4.09–4.04 (m, 1 H, H_2″_), 4.02–3.95 (m, 2 H, H_3″_, H_5′_), 3.40 (dd, *J*_H6′a-H6′b_ = 14.0, *J*_H6′a-H5′_ = 5.0 Hz, 1 H, H_6′a_), 3.33–3.31 (m, 1 H, H_6′b_), 3.26 (ddd, *J*_H5″a-H5″b_ = 16.2, *J*_H5″-H4″_ = 7.1 Hz, 2 H, H_5″_), 3.14–3.06 (m, 2 H, H_1×_), 1.78 (dt, *J*_H2×-H1×_ = 12.4, *J*_H2×-H3×_ = 7.7 Hz, 2 H, H_2×_), 1.45 (dt, *J*_H3×-H4×_ = *J*_H3×-H2×_ = 7.2 Hz, 2 H, H_3×_), 1.38–1.26 (m, 12 H), 0.90 (t, *J*_H10×-H9×_ = 7.0 Hz, 3 H, H_10×_); ^13^C NMR (125 MHz, MeOD) δ 166.1 (C_4_), 152.1 (C_2_), 142.3 (C_6_), 110.4 (C_1″_), 102.5 (C_5_), 92.2 (C_1′_), 84.8 (C_3′_), 80.0 (C_4″_), 78.6 (C_5′_), 76.2 (C_3″_), 75.4 (C_2′_), 73.9 (C_2″_), 71.0 (C_4′_), 53.3 (C_1×_), 45.5 (C_5″_), 44.5 (C_6′_), 33.02 (C_2×_), 30.6 (C_3×_), 30.5, 30.4, 30.3, 29.4, 24.7, 23.7, 14.4 (C_10×_); HRMS (TOF MS ES^+^) calcd for C_25_H_45_N_4_O_11_S^+^ (M + H)^+^ 609.2819, found 609.2820.

### 3.2. Enzyme Assays

The inhibitory activity of the synthesized compounds **1**–**5** was determined as described in Reference [32]. The compounds were evaluated on His-tagged MraY transferase purified from *Aquifex aeolicus* (MraY_AA_) prepared as previously described by Chung et al. [30]. The assays were performed as previously described by Stachyra et al. [44] in 96-well plates in a total reaction mixture of 100 µL containing 100 mM of Tris-HCl (pH 7.5), 40 mM MgCl_2_, 150 µM C_55_-P, 150 mM NaCl, 25 µM dansylated UDP-Mur*N*Ac-pentapeptide and 0.4% of *n*-dodecyl β-D-maltoside. The reaction was initiated by the addition of pure MraY_AA_ enzyme (10 µL, 0.036 mg/mL). Briefly, MraY catalyzed the formation of lipid I, displaying an apolar environment from polar and hydrosoluble dansylated-UDP-MurNAc-pentapeptide. This modification in the environment of the dansyl probe was accompanied by an enhancement (3.4 times) of fluorescence, as well as a shift of the maximum of fluorescence emission spectrum from 560 nm for the dansylated nucleotidic substrate to 530 nm for the dansylated lipid product (see Figure 2 in Stachyra et al. [44]. This property was exploited to develop an HTS assay [44] and to assess MraY activity by using an Enspire fluorescence microplate reader (Perkin-Elmer, Courtaboeuf, France). The fluorescence measurement was read every two minutes at 37 °C, under shaking during 60 min; the excitation wavelength and the emission wavelength were 340 nm and 530 nm, respectively. Experiments were performed in triplicate, and each experiment was repeated at least twice. In each case, the fluorescence of a control sample without enzyme was subtracted, initial velocity was calculated and percent inhibition was deduced. IC_50_ values were determined from plots of the percent inhibition versus the inhibitor concentration, and data were processed on Excel software.

### 3.3. Antibacterial Activity

Tests were made according to the procedure described in Reference [32], following EUCAST (European Committee on Antimicrobial Susceptibility testing)/CLSI (Clinical and Laboratory Standard Institute) recommended procedure [45]. This microtitration plate size allows direct detection of bacterial growth in a relatively small volume, without the use of a spectrophotometer or the addition of dyes. Molecules were solubilized in 100% DMSO (cell culture grade) at 20.48 mg/mL concentration and 40-fold diluted in MHB to reduce DMSO concentration in the antibacterial test, just before utilization. The MHB-diluted solutions were then serially two-fold diluted in MHB, at final concentration ranging from 128 to 1 µg/mL. Bacterial inoculums were prepared for each strain, resuspending isolated colonies from 18 h cultured plates. Equivalents of 0.5 Mac Farland turbidity standard (approximately 1.108 CFU/mL) were prepared in saline solution (NaCl 0.085%) and diluted 200-fold in MBH. The bacterial suspensions were then added to microplates containing the diluted molecules. Microtitration plates were incubated overnight at 37 °C. MICs were determined as the lowest dilution of product showing no visual turbidity.

### 3.4. Docking and MD Simulations

Ligands were docked into the crystal structures of the MraY_AA_ in complex with muraymycin D2 (PDB: 5CKR) and carbacaprazamycin (PDB: 6OYH) from CDOCKER program implemented in Biovia Discovery Studio 2016 [41] and according to the protocole described previously [32]. MD simulations were carried out on the 6OYH model using NAMD program [43] in Biovia Discovery studio 2021 as reported previously [32].

## 4. Conclusions

In this paper, we reported the synthesis of five new inhibitors of the bacterial MraY transferase displaying an aminoribosyl uridine scaffold substituted in 5′ position by various linkers (squaramide, diamides, amide or sulfonamide) bearing an identical decyl chain. Their straightforward synthesis involved classical transformations, such as amidation, peptidic coupling or sulfonylation reactions from a conveniently protected azidoribosyl uridine with an aminomethyl group at C-5′. Their biological activity was evaluated in vitro on purified MraY_AA_ and compared to that of related reference compounds with a urea, a *C*- or an *N*-triazole linker. All compounds revealed MraY inhibition, with IC_50_ ranging from 0.37 to 17.97 µM. The binding mode of the inhibitors was studied by docking experiments. Molecular dynamics studies revealed the crucial role of the diamide linker to stabilize the more active compound (*S*,*S*)-**3a** through interactions with N190 and H325, which are two invariant residues in the active site of the MraY enzymes [46]. These interactions promote an optimal orientation of the key fragments (aminoribosyl uridine and alkyl chain) within the hotspots of MraY_AA_ catalytic site and explain the improved activity of this compound compared to the reference urea **13**. The in cellulo evaluation of the synthetized inhibitors on different Gram-positive and Gram-negative bacterial strains showed no significant activity that can be explained by the too-short length of their alkyl chain. Further optimization of the lipophilic side chain is still required to increase the antibacterial activity.

## Figures and Tables

**Table 1:** Inhibitory activity of compounds **2**–**5** and **12**–**15** against MraY_AA_.

Compound	Linker	IC_50_ (µM) ^a^
Tunicamycin	-	0.026 ± 0.00
**12**	-	50.30 ± 3.27
**13**	Urea	1.93 ± 0.13
**14**	*C*-triazole	3.74 ± 0.11
**15**	*N*-triazole	2.14 ± 0.09
**2**	Squaramide	17.97 ± 0.04
(*S*,*S*)-**3a**	(*S*,*S*)-diamide	0.37 ± 0.01
(*R,R*)-**3b**	(*R*,*R*)-diamide	1.38 ± 0.05
**4**	Amide	6.49 ± 0.21
**5**	Sulfonamide	2.91 ± 0.13

^a^ Experiments were performed in triplicate, and each experiment was repeated at least twice, except for tunicamycin as a control that was tested twice. The background color corresponds to reference compounds already known while the lines with no background correspond to the new compounds described in the manuscript.

**Table 2:** Comparison of docking score in models 5CKR and 6OYH.

Compound	IC_50_	Docking Score ^a^	
	(µM)	5CKR	6OYH
Muraymycin D2	0.046 ^b^	103.6	ND ^c^
Carbacaprazamycin	0.104 ^d^	ND ^c^	92.13
**2**	17.97 ± 0.04	78.9	80.1
**4**	6.49 ± 0.21	80.3	73.7
**5**	2.91 ± 0.13	80.4	73.0
**13**	1.93 ± 0.13	79.2	80.0
(*R*,*R*)-**3b**	1.38 ± 0.05	87.3	83.3
(*S*,*S*)-**3a**	0.37 ± 0.01	89.1	86.0

^a^ Docking score obtained from -CDOCKER interaction energy. ^b^ Reference [41]. ^c^ ND: not determined. ^d^ Reference [31].

**Table 3:** MraY_AA_ binding site hotspots (PDB: 5CKR) for the aminoribosyl and linker moieties of hits.

	Compound	13	3a	3b	2	4	5
**Aminoribosyl moiety**	T75						
	N190						
	D193						
	G264						
	S268						
	K121						
	D118						
	D265						
**Linker moiety**	D193						
	H324						
	H325						
	L191						

Binding site hotspots were colored as follows: HS1 (green), HS2 (purple), HS3 (pink), HS5 (orange) and HS6 (gray). A detailed view of the interactions between MraY_AA_ and the hits is available in Supplementary Materials Figure S1.

**Table 4:** MraY_AA_ binding-site hotspots (PDB: 6OYH) for the aminoribosyl and linker moieties of hits.

	Compound	13	3a	3b	2	4	5
**Aminoribosyl moiety**	T75						
	N190						
	D193						
	G264						
	K121						
	D118						
	D265						
**Linker moiety**	N190						
	H325						
	L191						

Binding-site hotspots are colored as follows: HS1 (green), HS2 (purple), HS3 (pink), HS5 (orange) and HS6 (gray). A detailed view of the interactions between MraY_AA_ and the hits is available in Supplementary Materials Figure S2.

## Data Availability

The data presented in this study are available in Supplementary Material.

## Supplementary Materials

The following supporting information can be downloaded at: https://www.mdpi.com/article/10.3390/molecules27061769/s1. 1H and 13C spectra of all compounds. Table S1: Antibacterial activity of compounds **2**–**13** and reference compounds. Table S2: Distance measurement (Å) between hits and interacting residues of MraYAA binding site from docking experiments in model 5CKR and 6OYH. Figure S1: 2D diagram of ligands interactions in MraY_AA_ from docking experiments (PDB: 5CKR). Figure S2: 2D diagram of ligands interactions in MraY_AA_ from docking experiments (PDB: 6OYH). Figure S3: Time evolution of the distances between binding site residues of MraYAA and ligands atoms. Figure S4: 2D diagram of ligands interactions in MraY_AA_ from 50 ns MD simulations (PDB: 6OYH).

## Sample Availability

No samples of the compounds are available from the authors.
