# Supplementary material for: A Sub-Micromolar MraYAA Inhibitor with an Aminoribosyl Uridine Structure and a (S,S)-Tartaric Diamide: Synthesis, Biological Evaluation and Molecular Modeling
Source: Molecules. 2022 Mar 8;27(6):1769. doi: 10.3390/molecules27061769 (PMC8954382; doi:10.3390/molecules27061769)
Supplement: Supplementary file 1 [file molecules-27-01769-s001.zip › molecules-1605516-SI.pdf]

A Sub-Micromolar MraY$_{AA}$ Inhibitor with an Aminoribosyl Uridine Structure and a (*S*,*S*)-Tartaric Diamide: Synthesis, Biological Evaluation and Molecular Modeling

Martin Oliver[1], Laurent Le Corre[1], Mélanie Poinsot[1], Michaël Bosco[1], Hongwei Wan[1], Ana Amoroso[2], Bernard Joris[2], Ahmed Bouhss[3], Sandrine Calvet-Vitale[1,*] and Christine Gravier-Pelletier[1,*]

1 Université de Paris, Faculté des Sciences, UMR CNRS 8601, LCBPT, F-75006 Paris, France; martin.cg.oliver@gmail.com (M. O.); laurent.le-corre@u-paris.fr (L. L. C.); melanie.poinsot@u-paris.fr (M. P.); michael.bosco@u-paris.fr (M.B.); hongwei.wan@etu.parisdescartes.fr (H. W.)

2 Unité de Physiologie et Génétique Bactériennes, Centre d'Ingénierie des Protéines, Département des Sciences de la Vie, Université de Liège, Sart Tilman, B4000 - Liège 1-Belgique; amamoroso@ulg.ac.be (A. A.); bjoris@uliege.be (B. J.)

3 Université Paris-Saclay, INSERM U1204, Univ Evry, Structure-Activité des Biomolécules Normales et Pathologiques (SABNP), Evry-Courcouronnes, France; ahmed.bouhss@univ-evry.fr (A. B.)

* Correspondence: sandrine.calvet-vitale@u-paris.fr; Tel.: +33-176-534-246, christine.gravier-pelletier@u-paris.fr; Tel.: +33-176-534-228

ELECTRONIC SUPPLEMENTARY MATERIAL

Table of contents

^{1}H and ^{13}C Spectra 3

3-(Decylamino)-4-ethoxycyclobut-3-ene-1,2-dione 6 3

Methyl (2*S*,3*S*)-2,3-*O*-isopropylidene-4-(decylamino)-2,3-dihydroxy-4-oxobutanoate (*S*,*S*)-7a 4

Squaramide 8 6

(*S*,*S*)-Diamide 9a 7

(*R*,*R*)-Diamide 9b 8

Amide 10 9

Sulfonamide 11 10

Squaramide 2 11

***(S,S)*-Diamide 3a** 12

***(R,R)*-Diamide 3b** 13

Amide 4 14

Sulfonamide 5 15

Table S1. Antibacterial activity of compounds 2-5, 13 and reference compounds.. 16

Table S2: Distance measurement (Å) between hits and interacting residues of MraY$_{AA}$ binding site from docking experiments in model 5CKR and 6OYH.. 16

Figure S1. 2D-diagram of ligands interactions in MraY$_{AA}$ from docking experiments (PDB: 5CKR).. 17

Figure S2. 2D-diagram of ligands interactions in MraY$_{AA}$ from docking experiments (PDB: 6OYH).. 18

Figure S3. Time evolution of the distances between binding site residues of MraY$_{AA}$ and ligands atoms. 19

Figure S4. 2D-diagram of ligands interactions in MraY$_{AA}$ from 50 ns MD simulations (PDB: 6OYH)... 20

^{1}H and ^{13}C Spectra

3-(Decylamino)-4-ethoxycyclobut-3-ene-1,2-dione 6

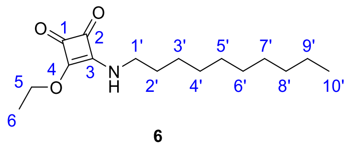

6

^{1}H NMR, 500 MHz ($CDCl_3$)

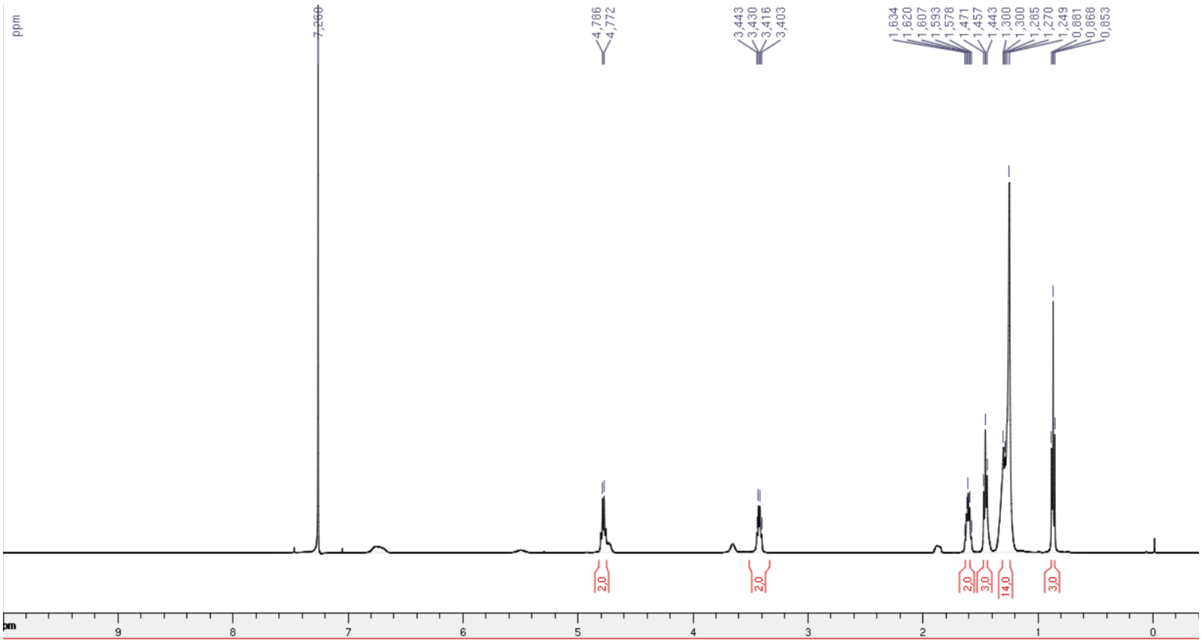

^{13}C NMR, 125 MHz ($CDCl_3$)

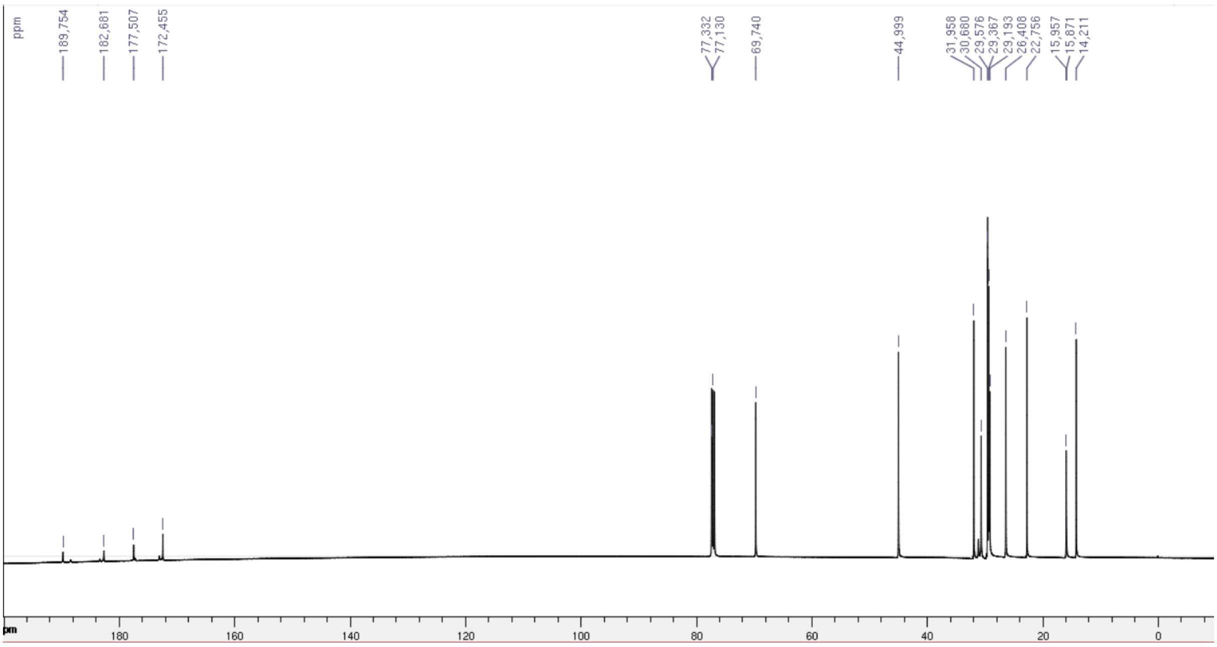

Methyl (2*S*,3*S*)-2,3-*O*-isopropylidene-4-(decylamino)-2,3-dihydroxy-4-oxobutanoate (*S*,*S*)-7a

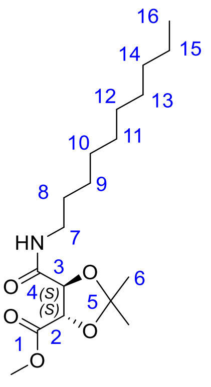

(*S*,*S*)-7a

^{1}H NMR, 500 MHz ($CDCl_3$)

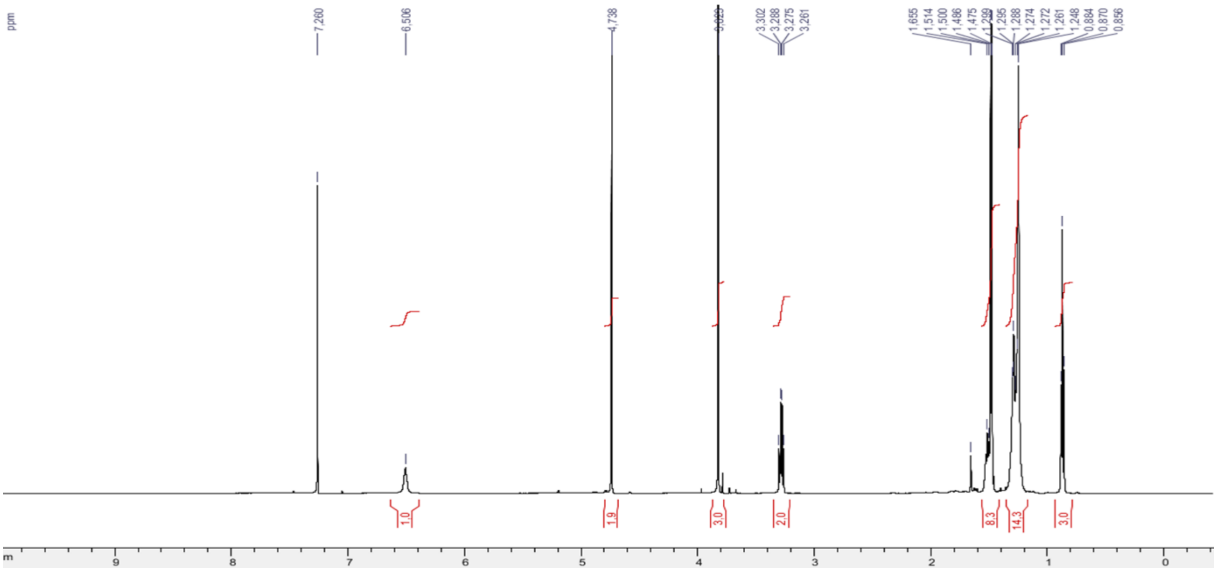

^{13}C NMR, 125 MHz ($CDCl_3$)

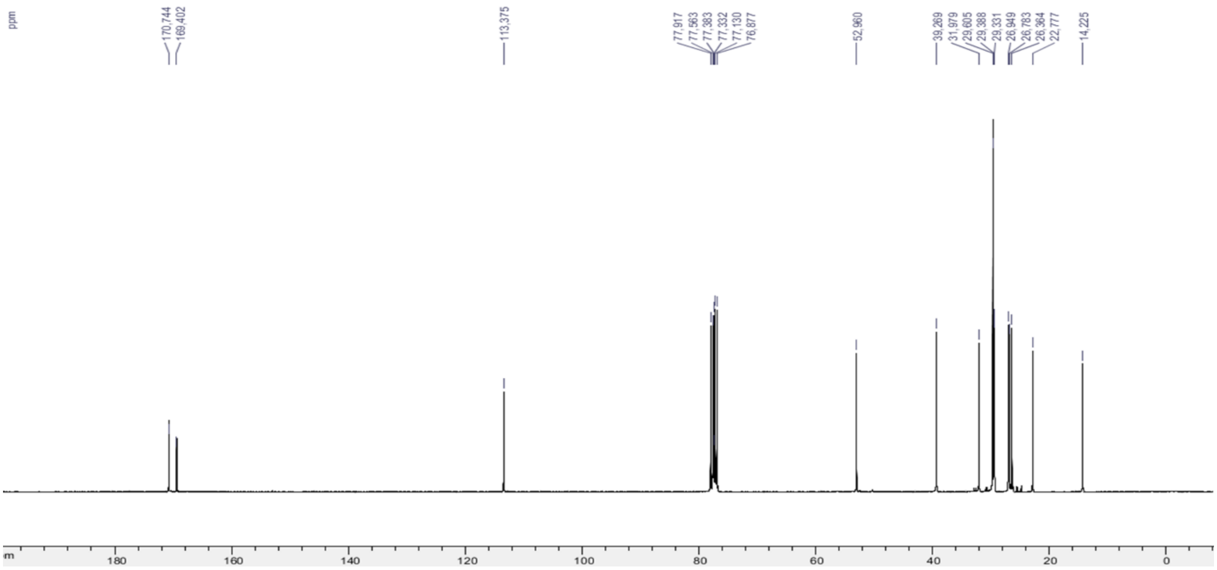

Methyl (2R,3R)-2,3-*O*-isopropylidene-4-(decylamino)-2,3-dihydroxy-4-oxobutanoate (R,*R*)-7b

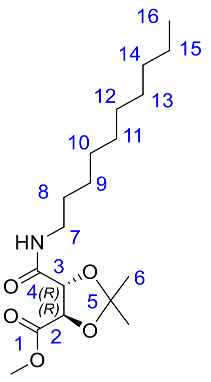

(*R*,*R*)-7b

^{1}H NMR, 500 MHz ($CDCl_3$)

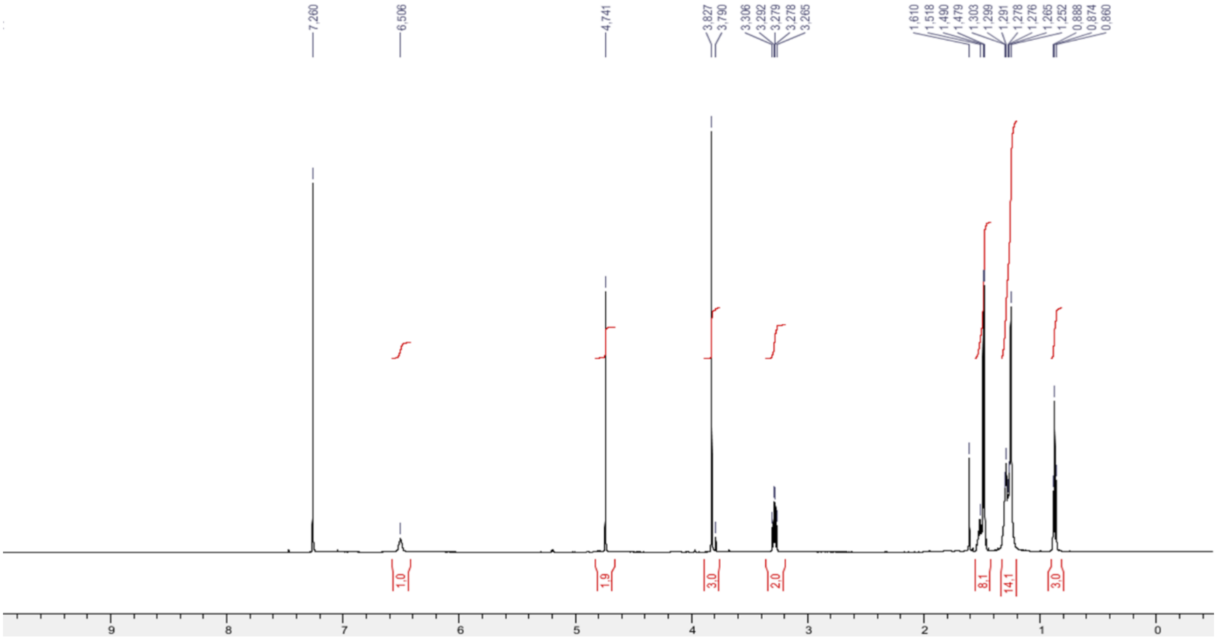

^{13}C NMR, 125 MHz ($CDCl_3$)

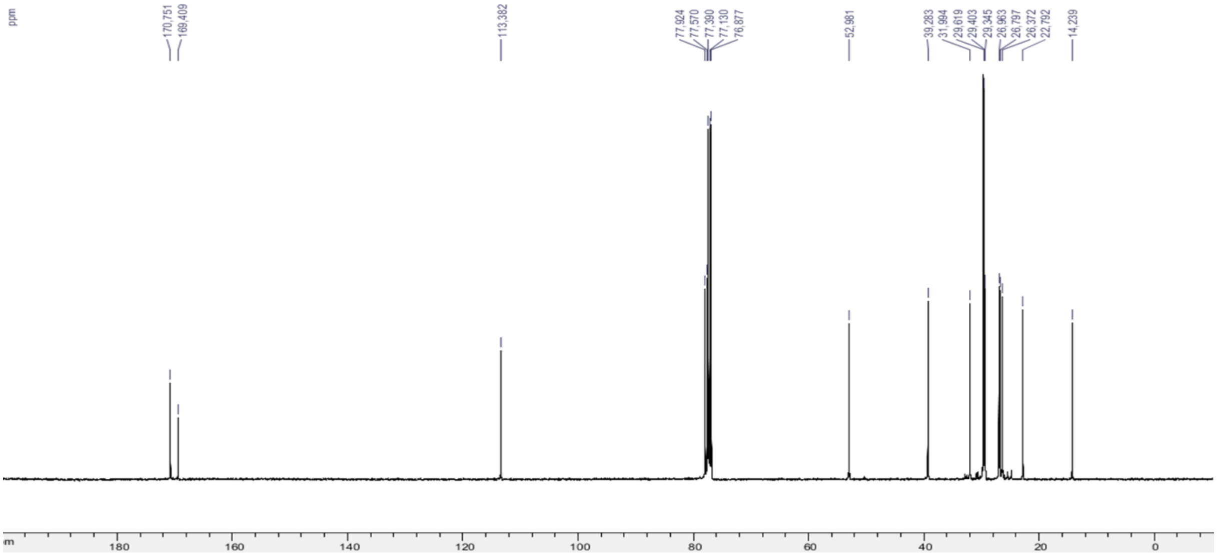

Squaramide 8

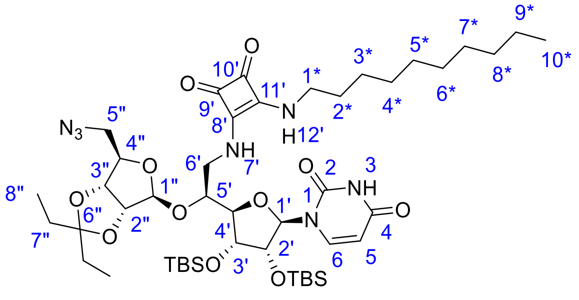

8

^{1}H NMR, 500 MHz ($CDCl_3$)

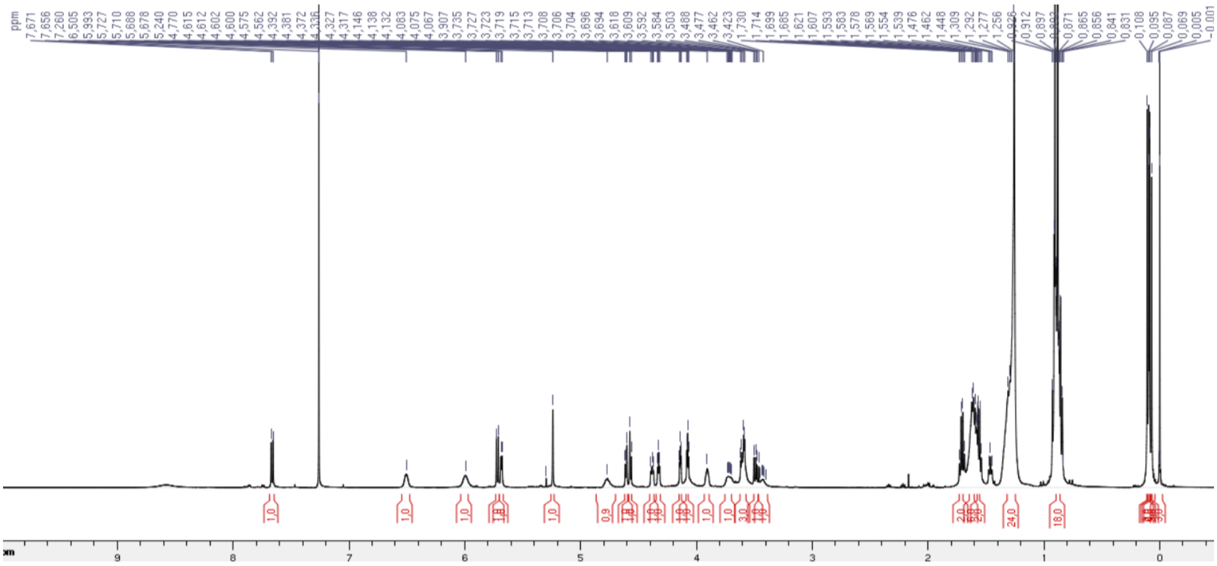

^{13}C NMR, 125 MHz ($CDCl_3$)

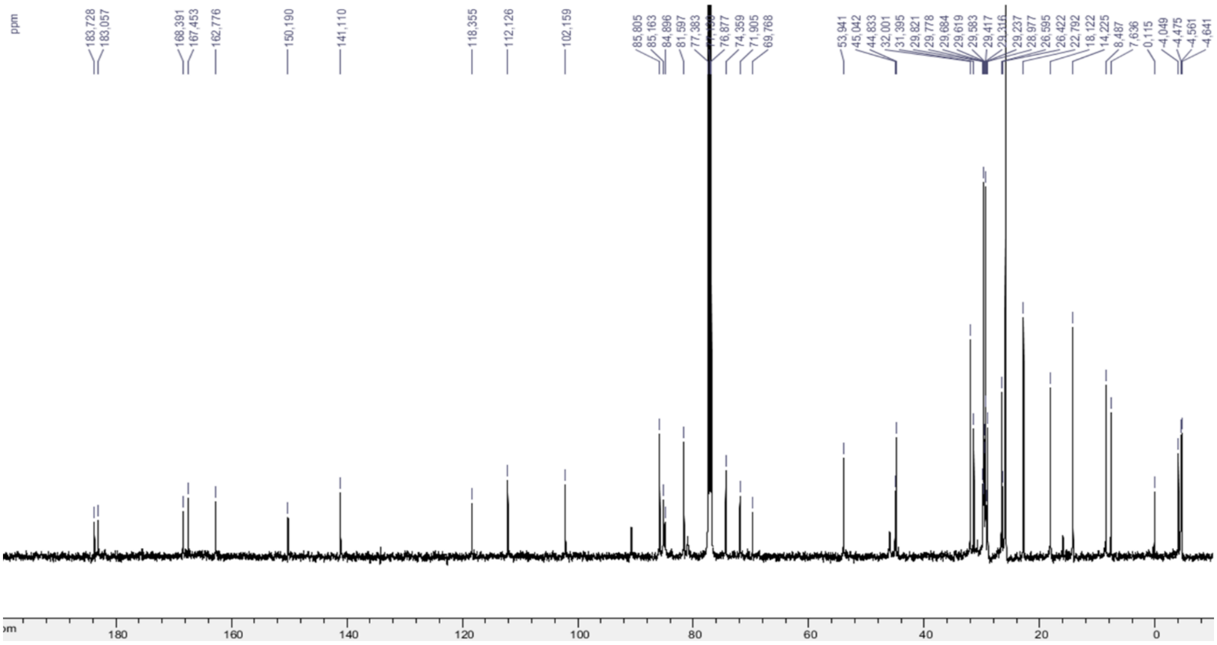

(*S*,*S*)-Diamide 9a

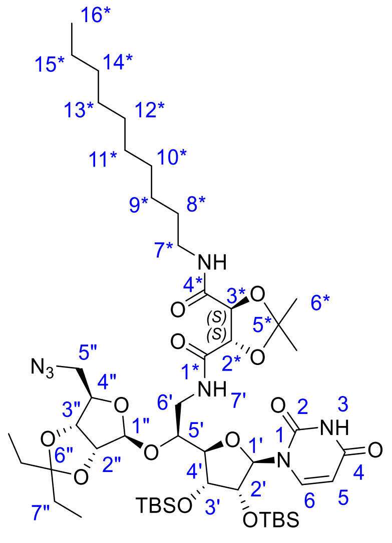

(*S*,*S*)-9a

^{1}H NMR, 500 MHz ($CDCl_3$)

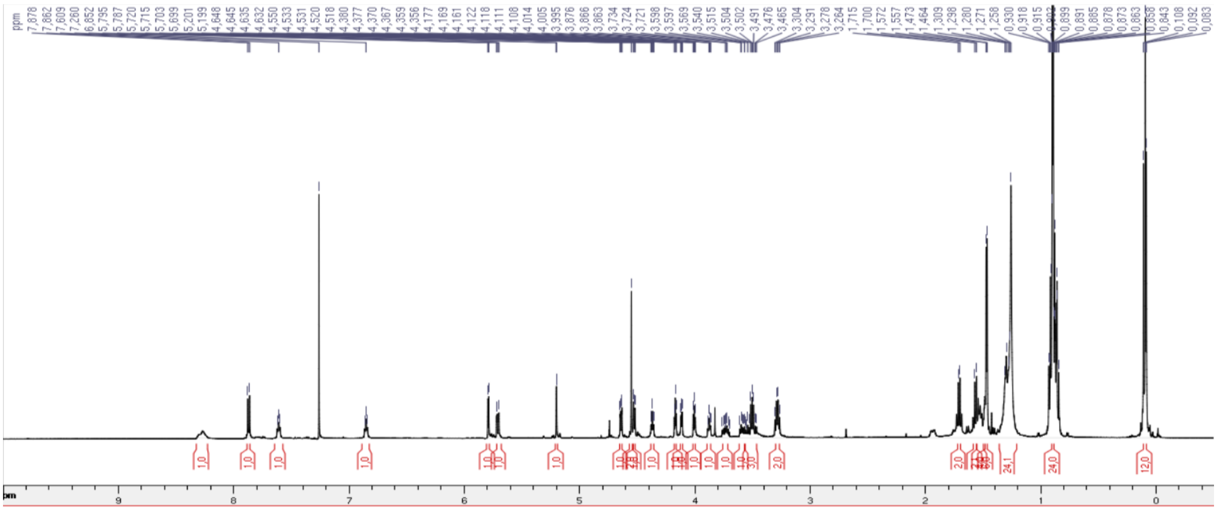

^{13}C NMR, 125 MHz ($CDCl_3$)

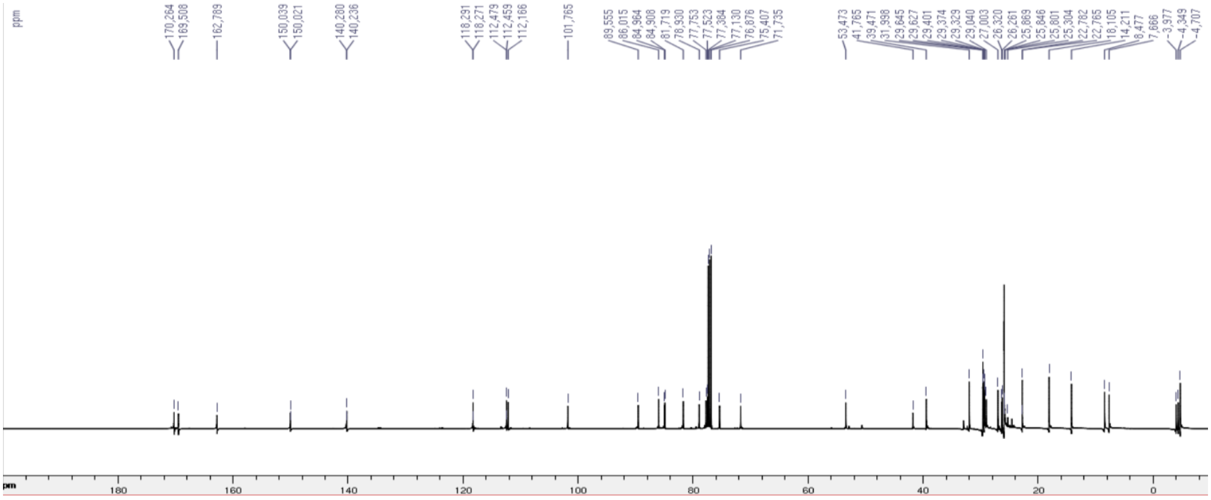

(*R*,*R*)-Diamide 9b

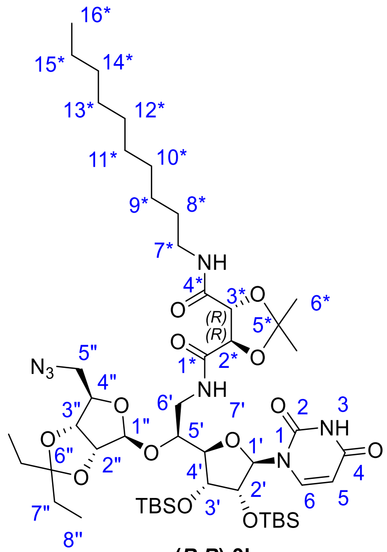

(*R*,*R*)-9b

^{1}H NMR, 500 MHz (CDCl$_3$)

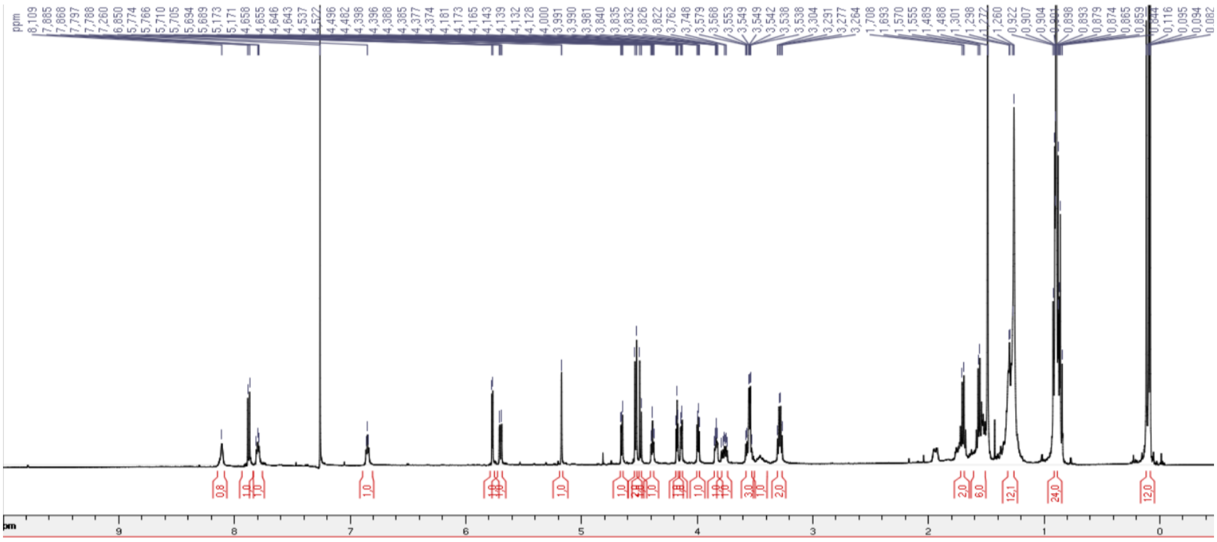

^{13}C NMR, 125 MHz (CDCl$_3$)

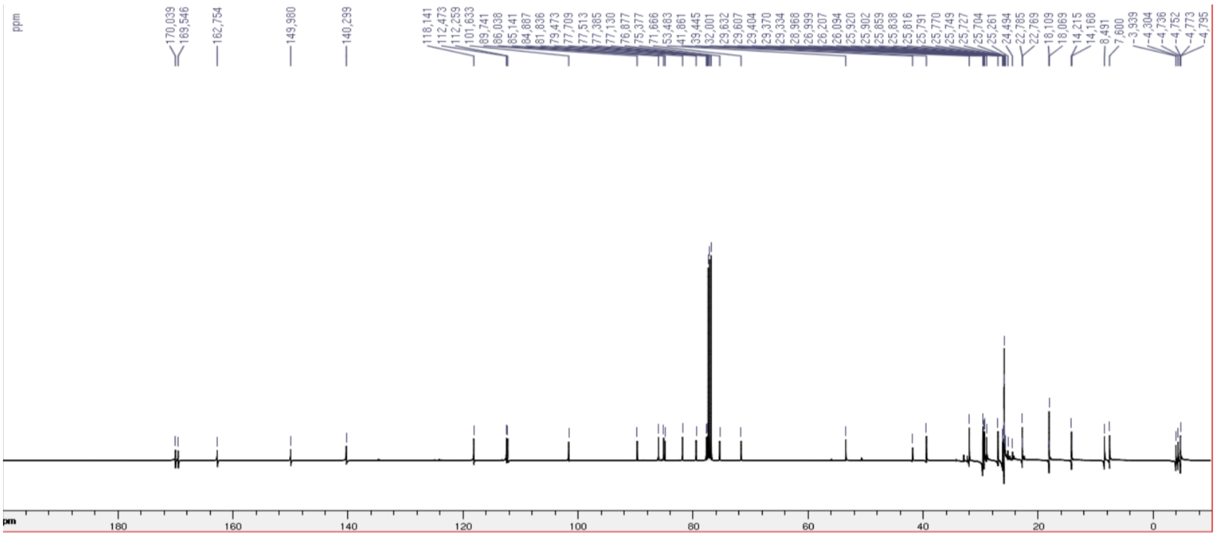

Amide 10

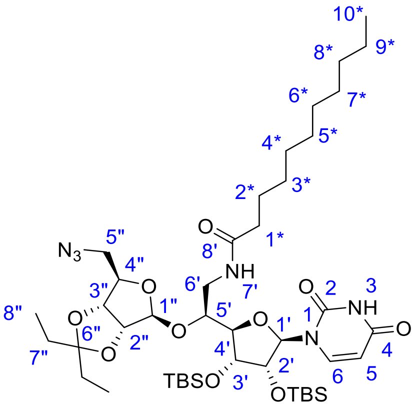

10

^{1}H NMR, 500 MHz ($CDCl_3$)

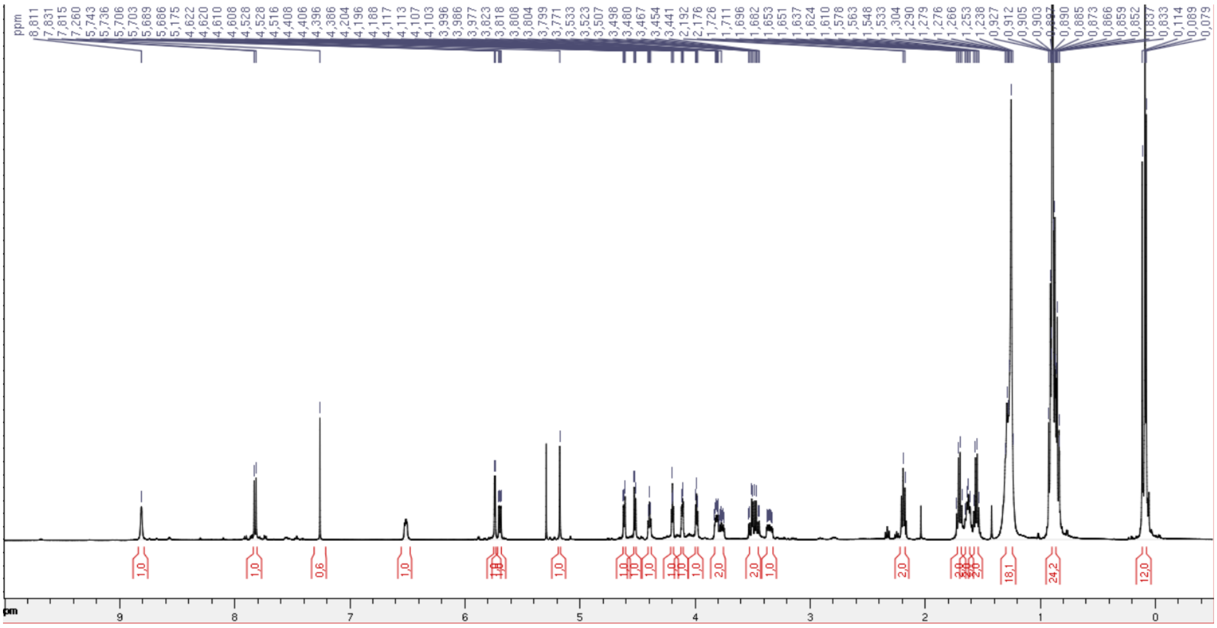

^{13}C NMR, 125 MHz (CDCl3)

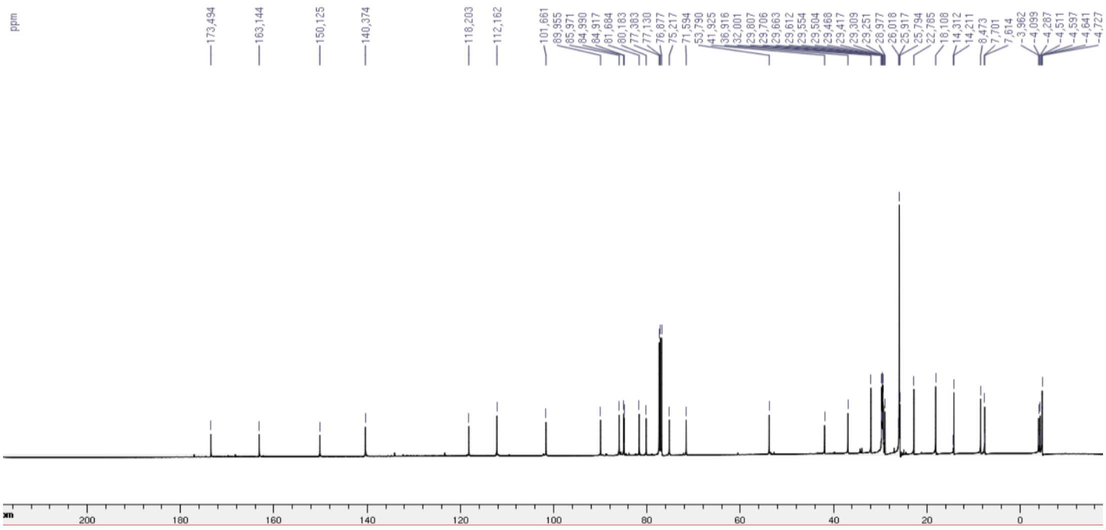

Sulfonamide 11

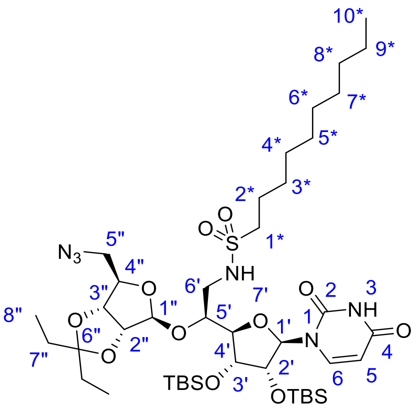

11

^{1}H NMR, 500 MHz (CDCl$_3$)

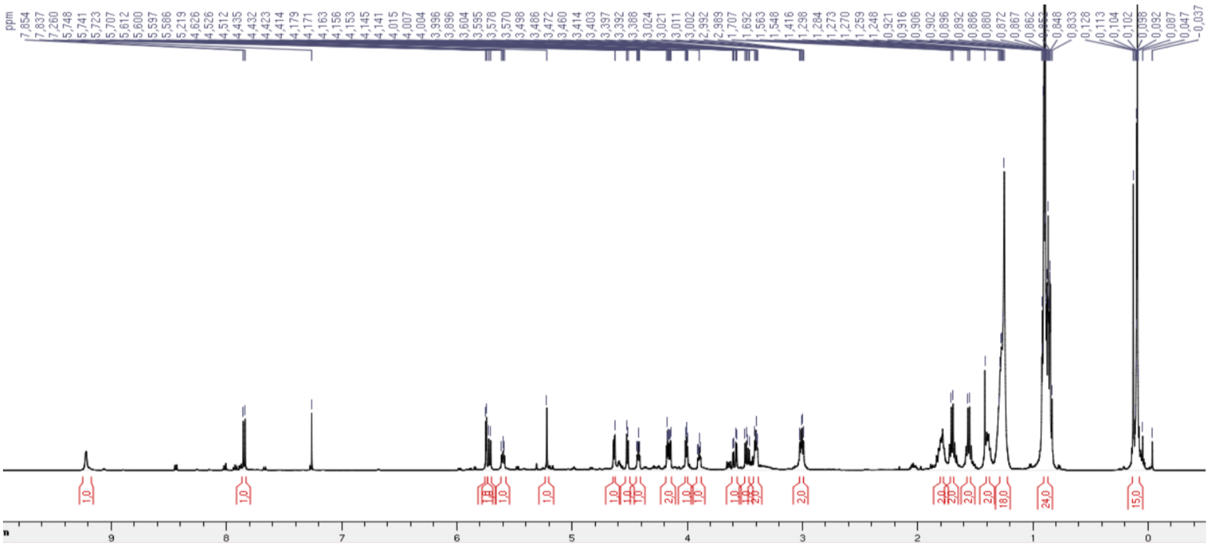

^{13}C NMR, 125 MHz (CDCl$_3$)

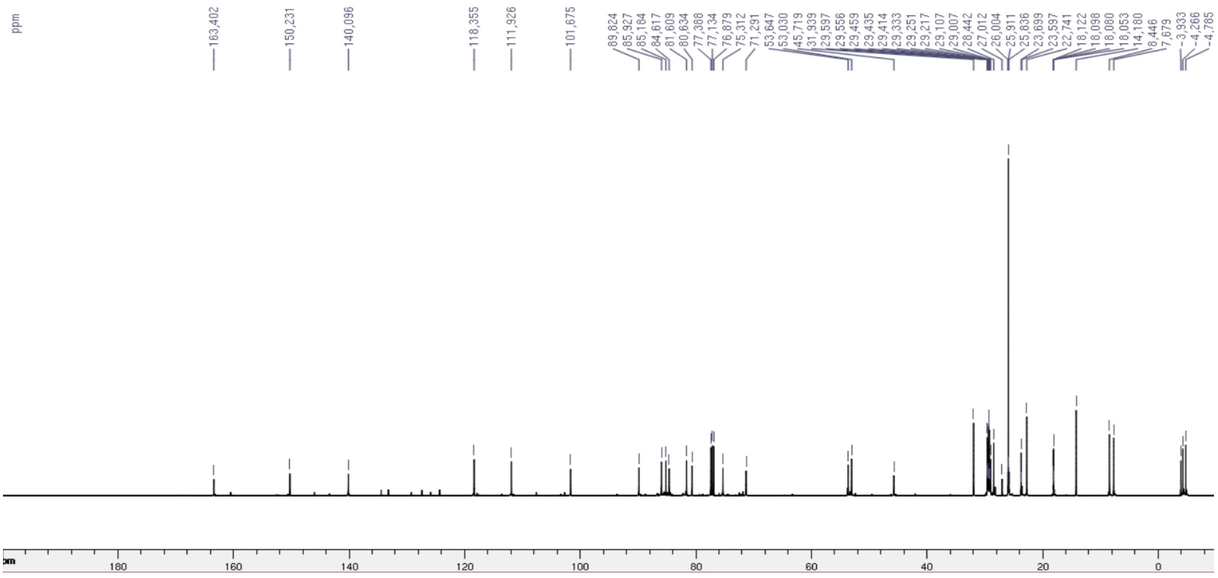

Squaramide 2

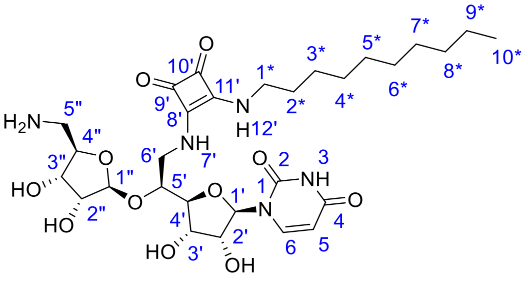

2

^{1}H NMR, 500 MHz (MeOD)

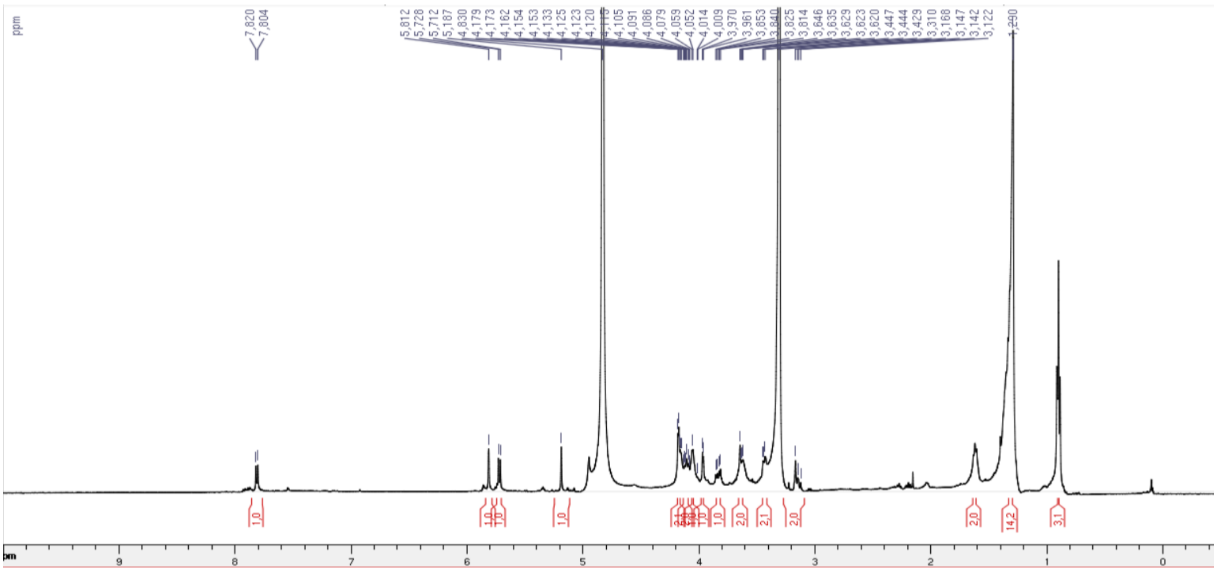

^{13}C NMR, 125 MHz (MeOD)

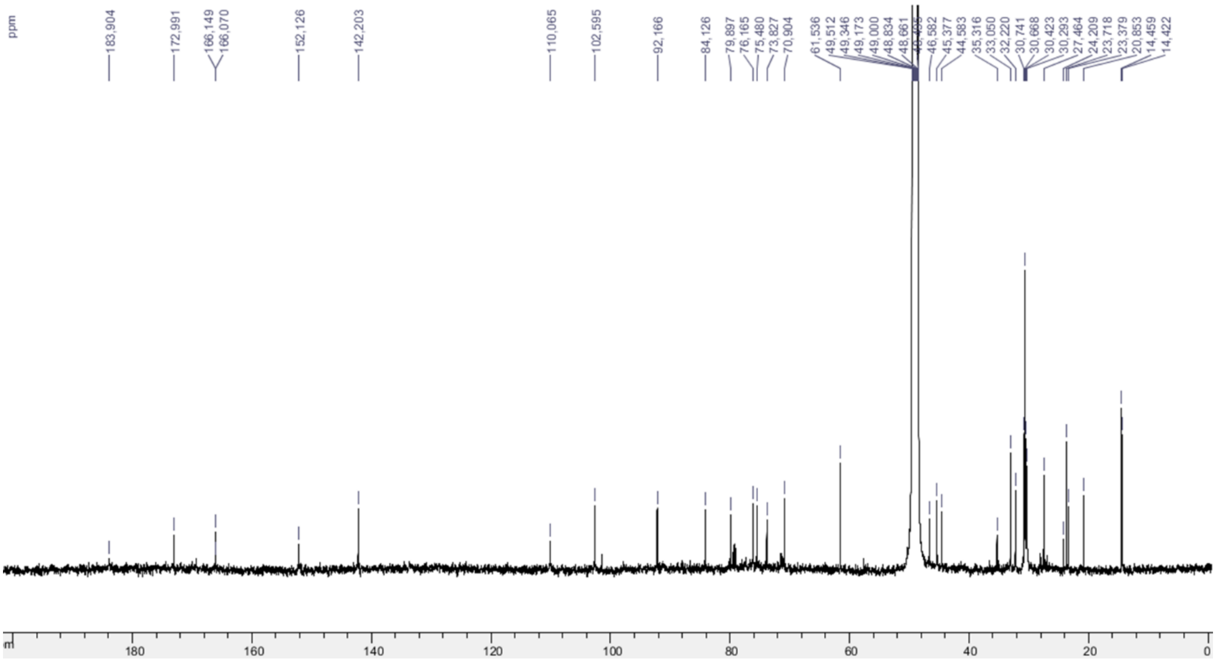

(S,S)-Diamide 3a

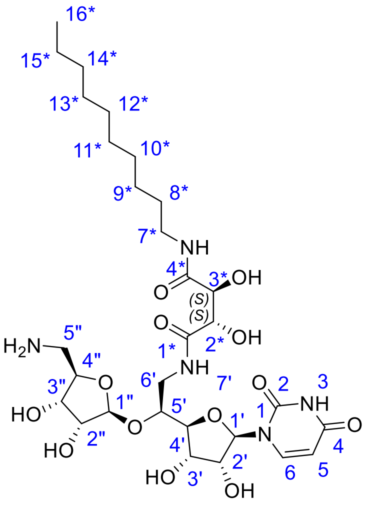

(*R*,*R*)-3a

^{1}H NMR, 500 MHz (MeOD)

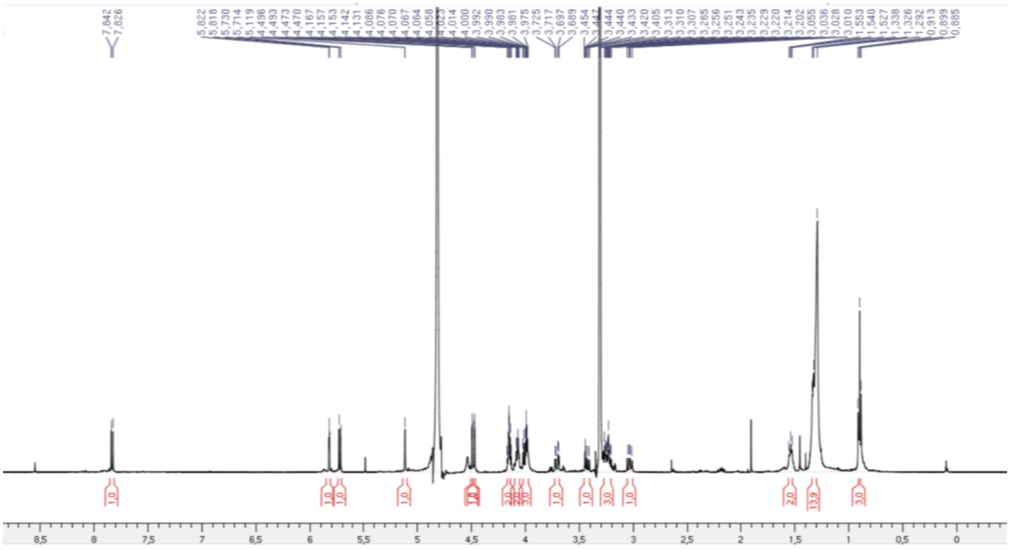

^{13}C NMR, 125 MHz (MeOD)

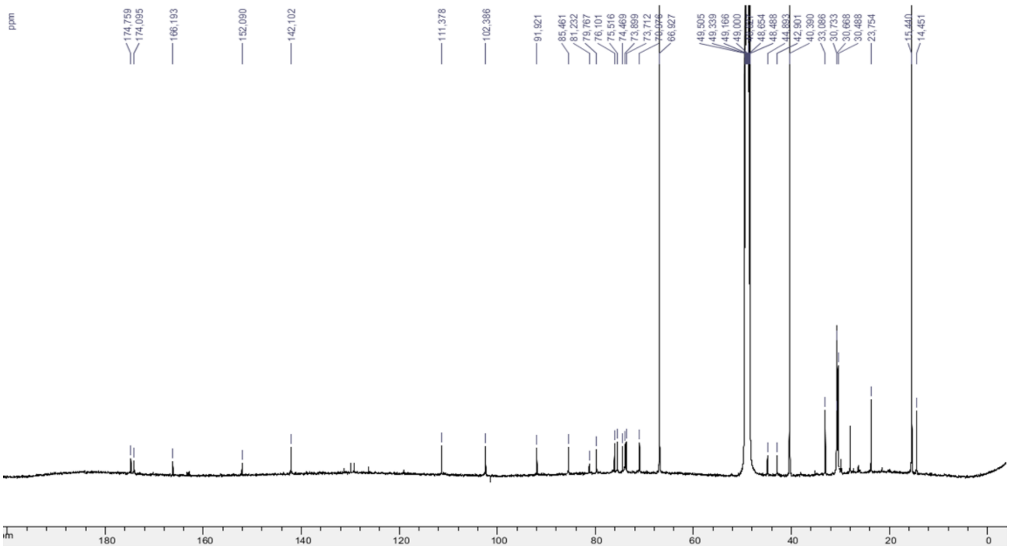

(R,R)-Diamide 3b

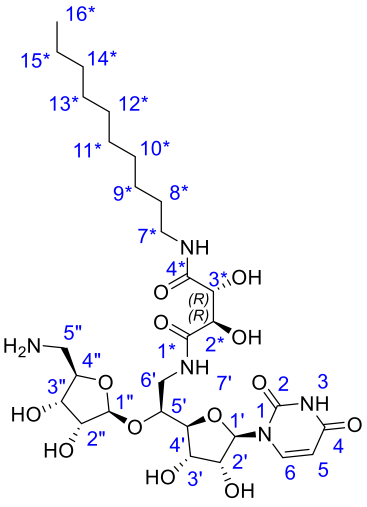

(***R***,***R***)-**3**

^{1}H NMR, 500 MHz (MeOD)

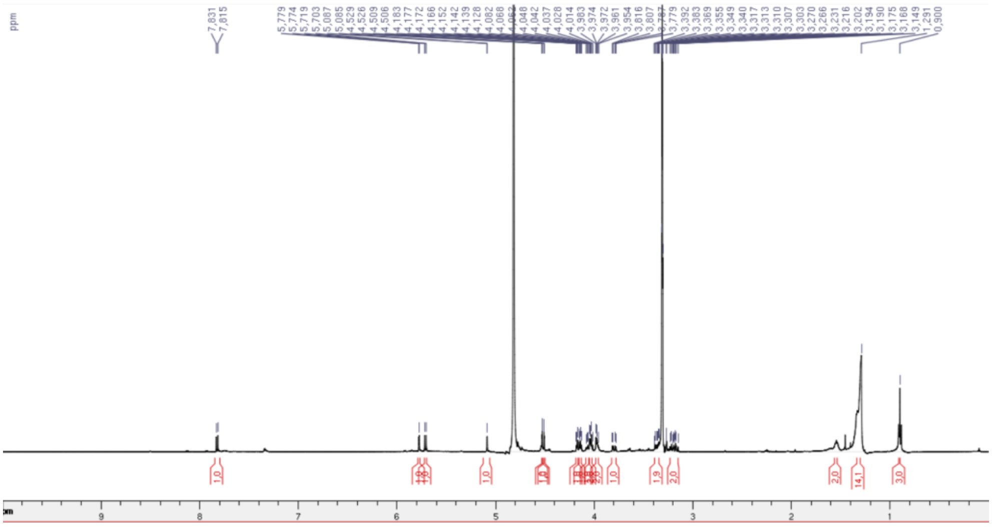

^{13}C NMR, 125 MHz (MeOD)

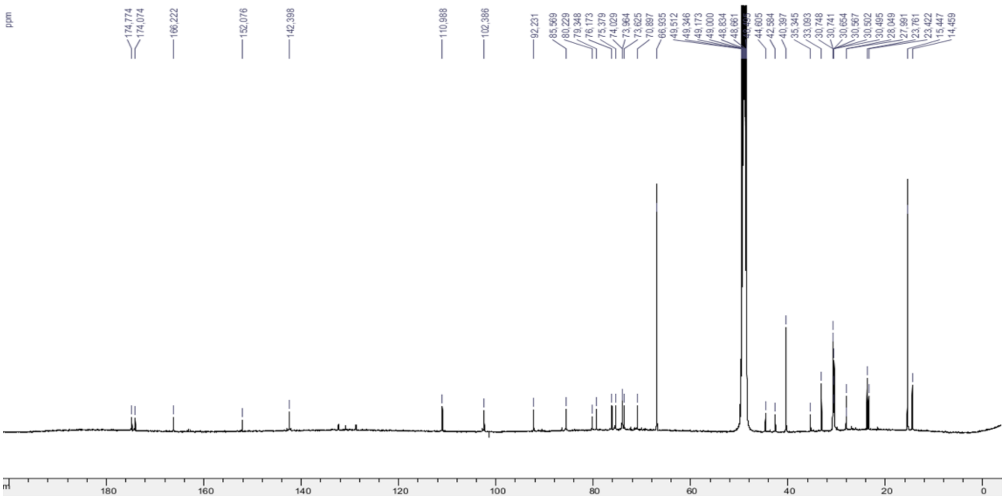

Amide 4

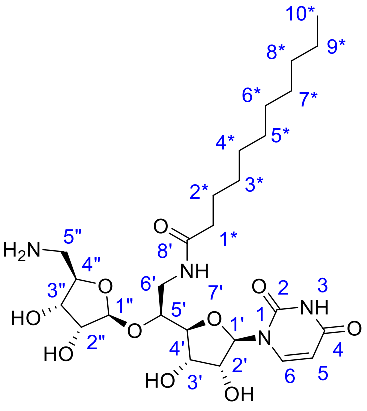

4

^{1}H NMR, 500 MHz (MeOD)

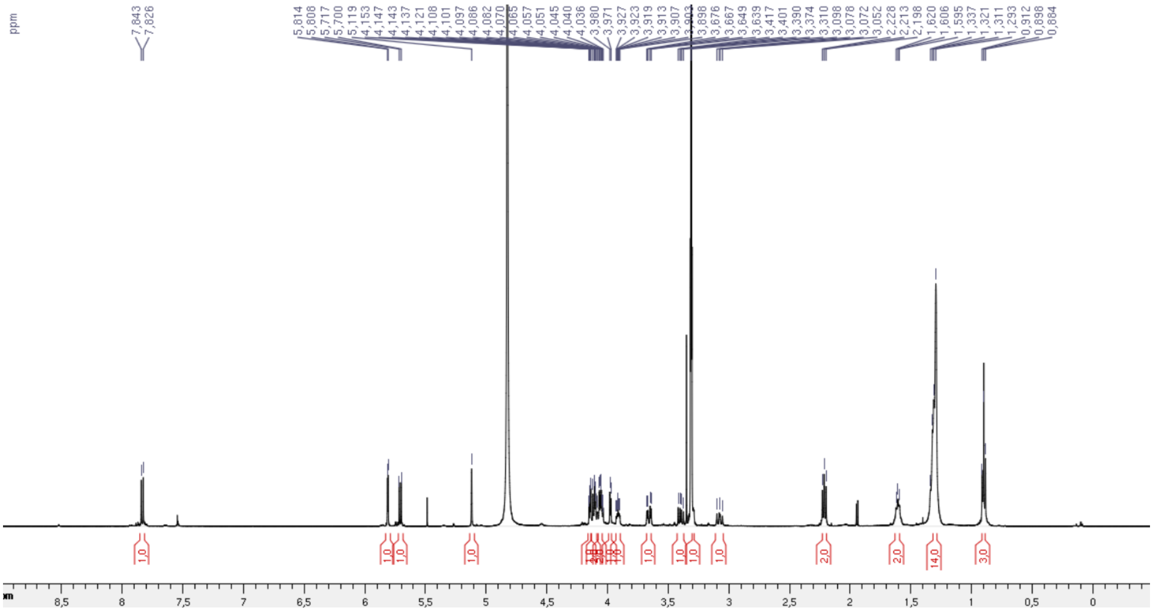

^{13}C NMR, 125 MHz (MeOD)

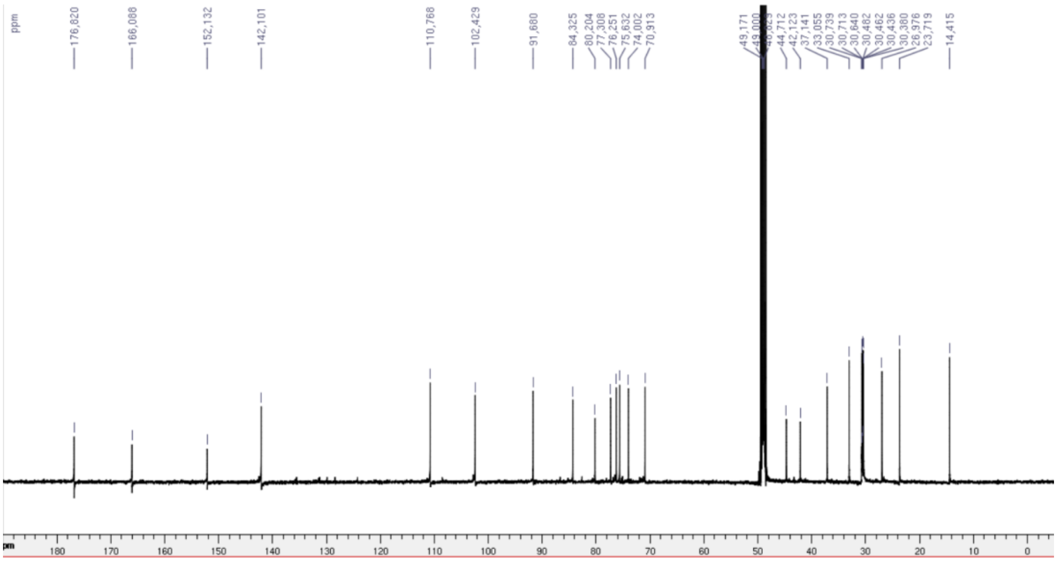

Sulfonamide 5

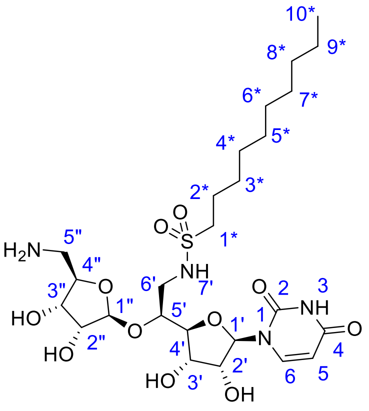

5

^{1}H NMR, 500 MHz (MeOD)

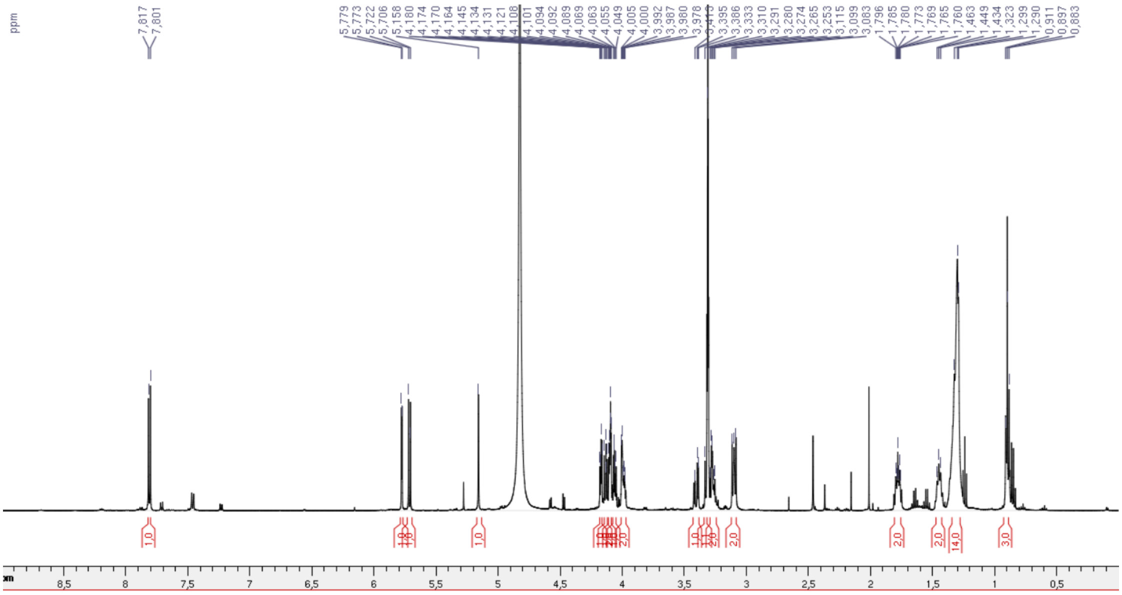

^{13}C NMR, 125 MHz (MeOD)

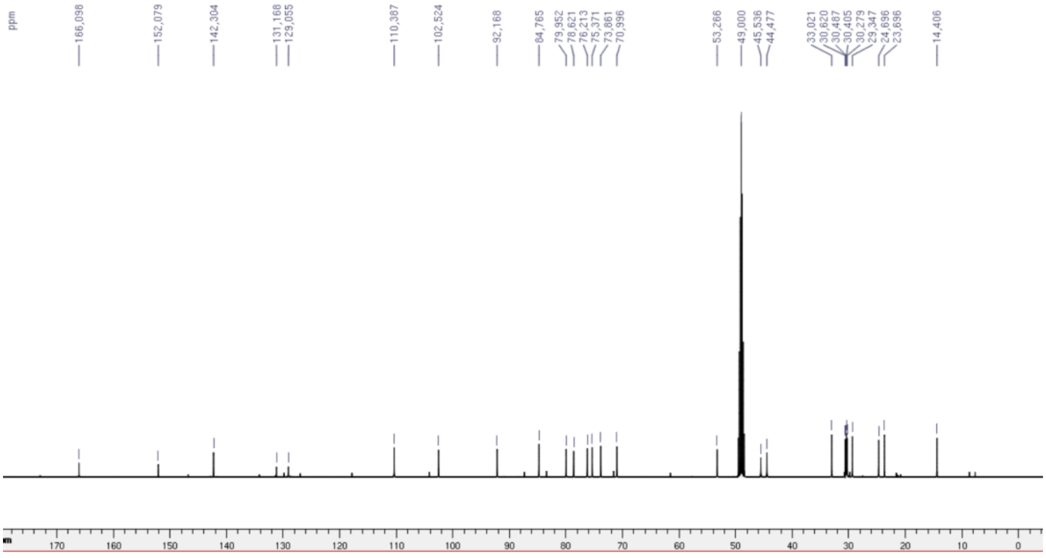

Table S1. Antibacterial activity of compounds **2-5**, **13** and reference compounds.

**Compound**	**Linker**	**CMI (µg/mL)**					
		Gram -			Gram +		
		Escherichia coli ATCC 8730	*Citrobacter freundii* ATCC 8090	*Pseudomonas aeruginosa* ATCC 27853	*Staphylococcus aureus* ATCC 25923	*Staphylococcus aureus MRSA* ATCC 43300	*Enterococcus faecium* ATCC 19434
**Piperacillin**		4	4	8	4	>128	4
**Vancomycin**		-	-	-	1	1	0.5
**13**	Urea	>128	>128	>128	>128	>128	>128
**2**	Squaramide	>128	>128	>128	>128	>128	>128
(*S,S*)-**3a**	(*S,S*)-Diamide	>128	>128	>128	128	>128	>128
(*R,R*)-**3b**	(*R,R*)-Diamide	>128	>128	>128	128	>128	>128
**4**	Amide	>128	>128	>128	128	128	128
**5**	Sulfonamide	>128	>128	>128	>128	>128	>128

Table S2: Distance measurement (Å) between hits and interacting residues of MraY$_{AA}$ binding site from docking experiments in model 5CKR and 6OYH.

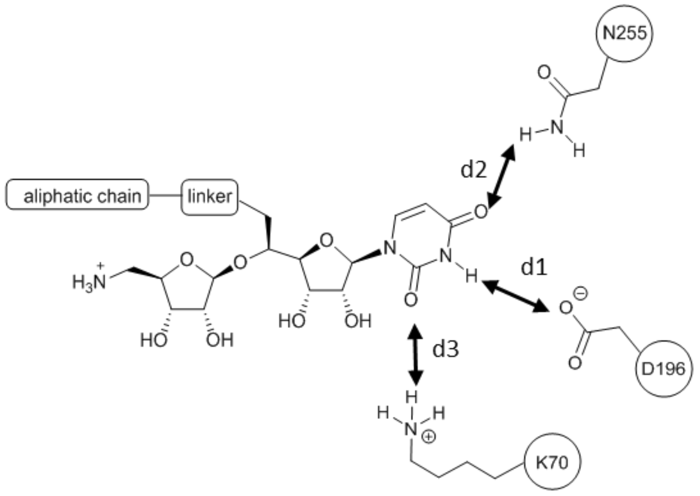

**Compound**	**IC$_{50}$**	**d1**		**d2**		**d3**	
		5CKR	6OYH	5CKR	6OYH	5CKR	6OYH
**13**	1.93 ± 0.13	1.90	2.00	1.94	2.21	1.69	-[a]
**2**	17.97 ± 0.04	1.86	1.92	2.44	1.84	1.69	2.43
(*S,S*)-**3a**	0.37 ± 0.01	1.85	1.85	2.34	2.29	1.79	1.65
(*R,R*)-**3b**	1.38 ± 0.05	1.87	1.95	2.43	2.40	1.66	1.73
**4**	6.49 ± 0.21	2.04	1.94	1.84	2.48	-	3.00
**5**	2.91 ± 0.13	1.87	1.94	2.46	2.12	2.70	-[a]

[a] not observed.

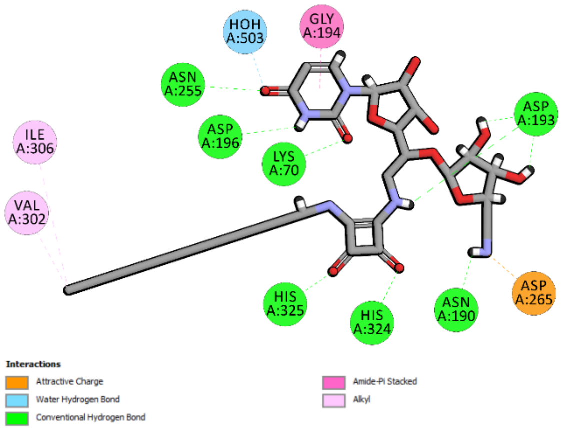

2

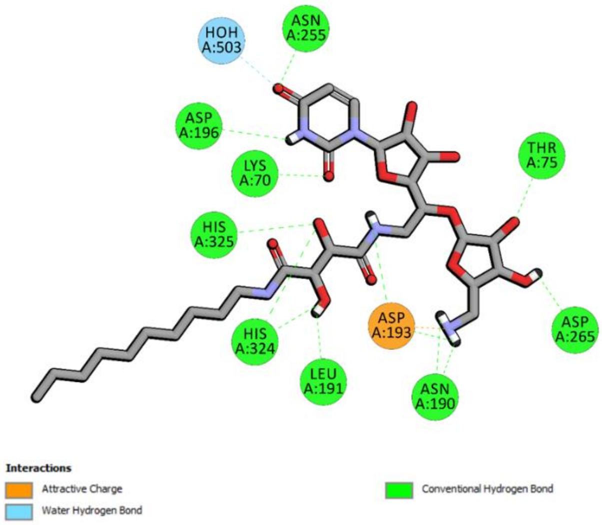

3a

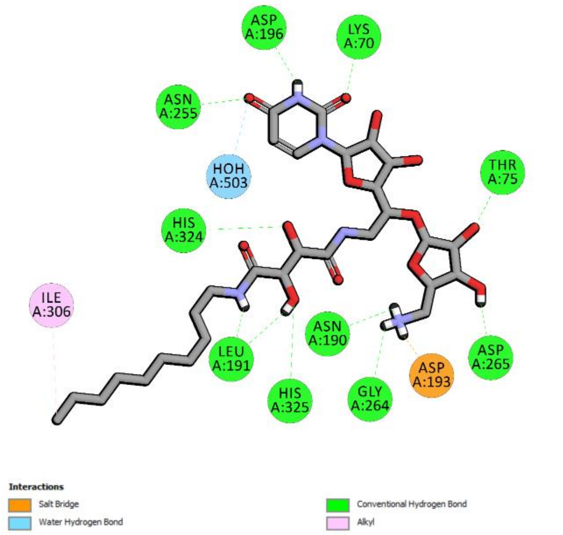

3b

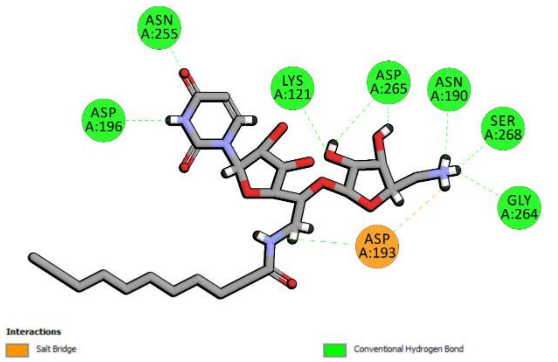

4

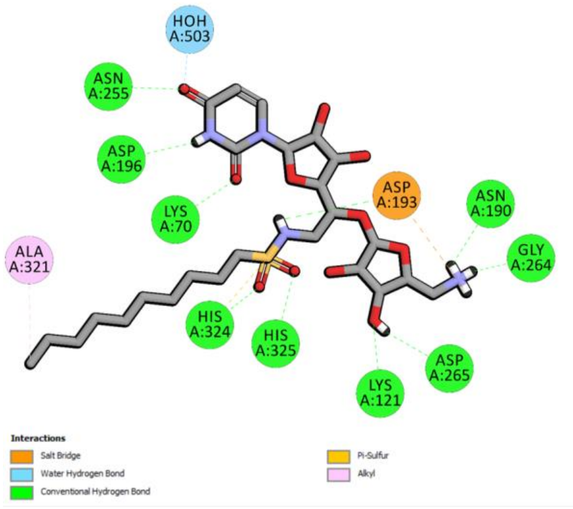

5

Figure S1. 2D-diagram of ligands interactions in MraYAA from docking experiments (PDB: 5CKR)

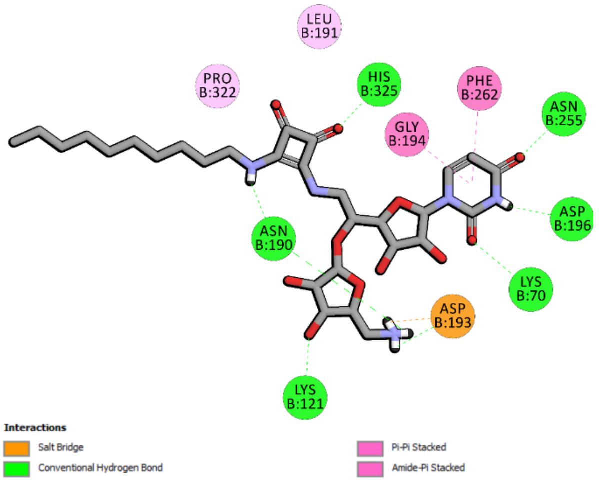

2

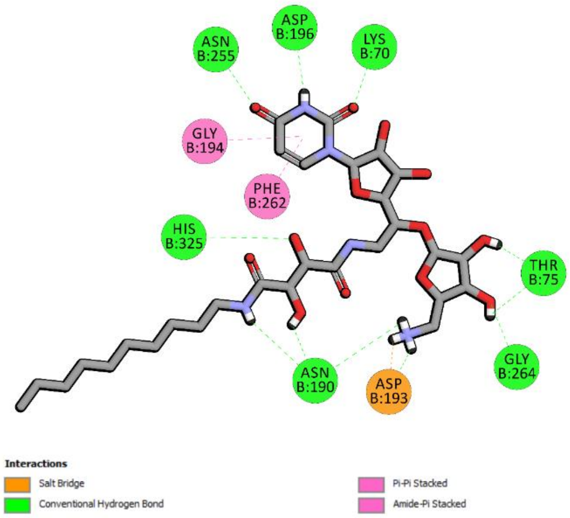

(*S*,*S*)-**3a**

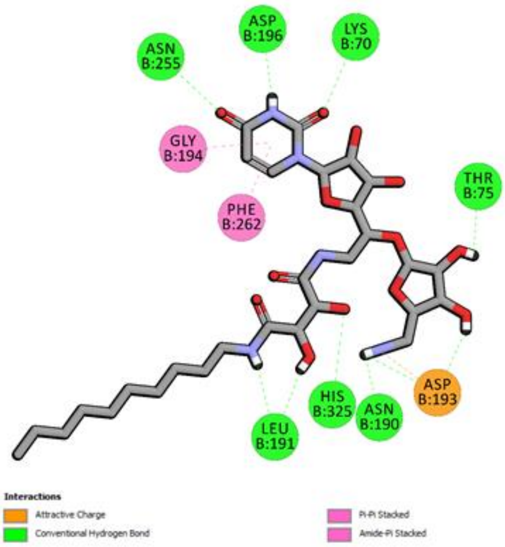

(*R*,*R*)-**3b**

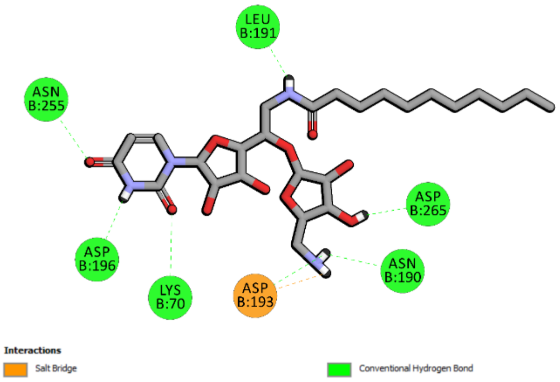

4

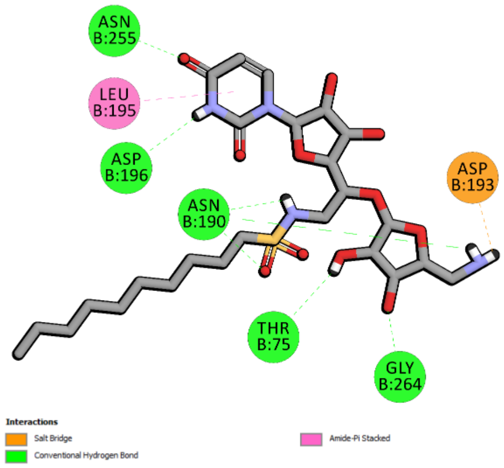

5

Figure S2. 2D-diagram of ligands interactions in MraYAA from docking experiments (PDB: 6OYH)

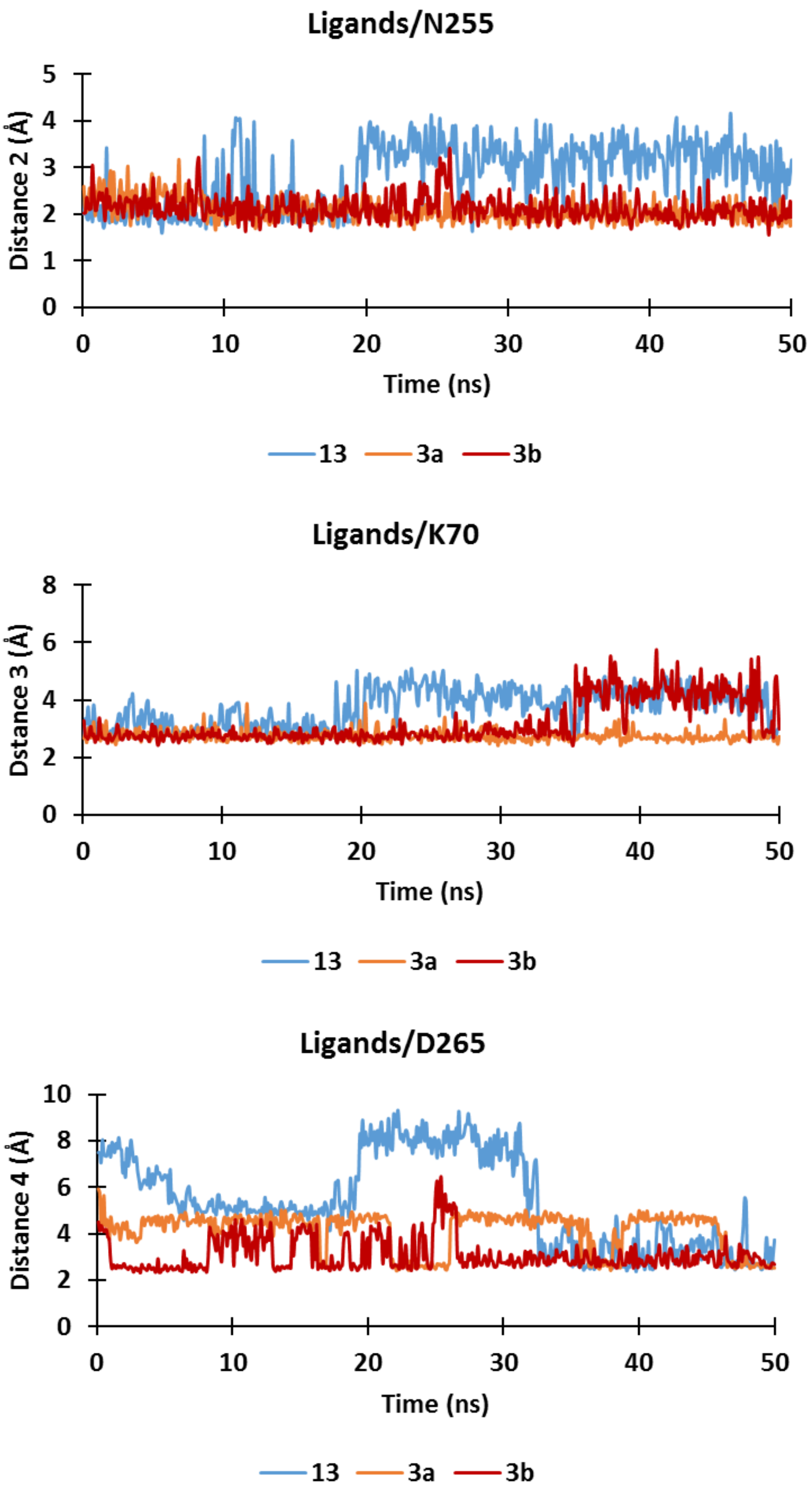

Figure S3. Time evolution of the distances between binding site residues of $MraY_{AA}$ and ligands atoms. A) Distance d2 between the NH_2 group of N255 and the NH group of the uridine moiety. B) Distance d3 between the centroid of the amino group of K70 and O40 atom of the uridine moiety. C) Distance d4 between the OD1 atom of D265 and the centroid of the amino group of ligands.

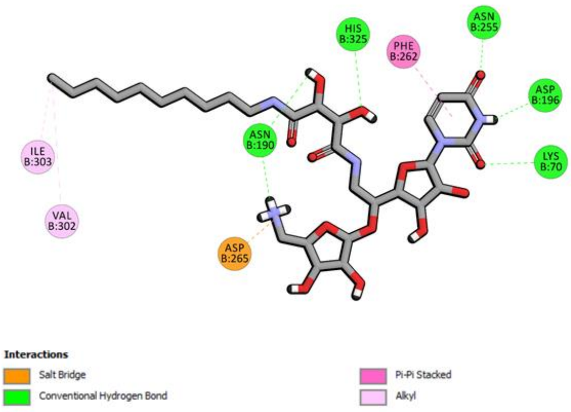

(*S*,*S*)-**3a**

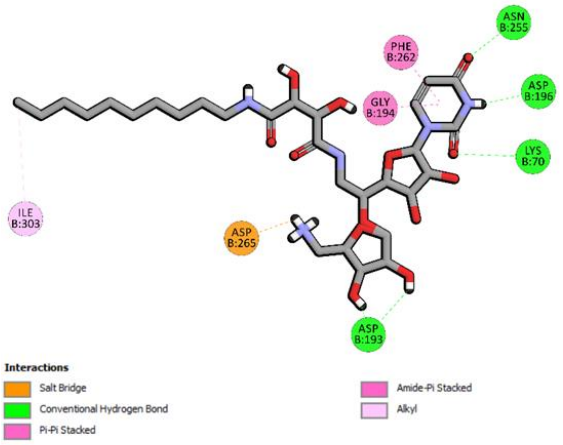

(*R*,*R*)-**3b**

Figure S4. 2D-diagram of ligands interactions in MraY$_{AA}$ from 50 ns MD simulations (PDB: 6OYH)
